# The role of specialized hospital units in infection and mortality risk reduction among patients with hematological cancers

**DOI:** 10.1371/journal.pone.0211694

**Published:** 2019-03-20

**Authors:** Raïsa Carmen, Galit B. Yom-Tov, Inneke Van Nieuwenhuyse, Bram Foubert, Yishai Ofran

**Affiliations:** 1 Department of Decision Sciences and Information Management, Faculty of Business and Economics, KU Leuven, Brussels Campus, Brussel, Belgium; 2 Faculty of Industrial Engineering and Management, Technion, Haifa, Israel; 3 Faculty of Business Economics, Hasselt University, Campus Diepenbeek, Diepenbeek, Belgium; 4 Department of Marketing and Supply Chain Management, Maastricht University, Maastricht, The Netherlands; 5 Department of Hematology and Bone Marrow Transplantation, Rambam Health Care Campus and Bruce Rappaport Faculty of Medicine, Technion, Haifa, Israel; Universidade Nova de Lisboa Instituto de Higiene e Medicina Tropical, PORTUGAL

## Abstract

**Motivation:**

Patients with hematological malignancies are susceptible to life-threatening infections after chemotherapy. The current study aimed to evaluate whether management of such patients in dedicated inpatient and emergency wards could provide superior infection prevention and outcome.

**Methods:**

We have developed an approach allowing to retrieve infection-related information from unstructured electronic medical records of a tertiary center. Data on 2,330 adults receiving 13,529 chemotherapy treatments for hematological malignancies were identified and assessed. Infection and mortality hazard rates were calculated with multivariate models. Patients were randomly divided into 80:20 training and validation cohorts. To develop patient-tailored risk-prediction models, several machine-learning methods were compared using area under the curve (AUC).

**Results:**

Of the tested algorithms, the probit model was found to most accurately predict the evaluated hazards and was implemented in an online calculator. The infection-prediction model identified risk factors for infection based on patient characteristics, treatment and history. Observation of patients with a high predicted infection risk in general wards appeared to increase their infection hazard (p = 0.009) compared to similar patients observed in hematology units. The mortality-risk model demonstrated that for infection events starting at home, admission through hematology services was associated with a lower mortality hazard compared to admission through the general emergency department (p = 0.007). Both models show that dedicated hematological facilities and emergency services improve patient outcome post-chemotherapy. The calculated numbers needed to treat were 30.27 and 31.08 for the dedicated emergency and observation facilities, respectively. Infection hazard risks were found to be non-monotonic in time.

**Conclusions:**

The accuracy of the proposed mortality and infection risk-prediction models was high, with the AUC of 0.74 and 0.83, respectively. Our results demonstrate that temporal assessment of patient risks is feasible. This may enable physicians to move from one-point decision-making to a continuous dynamic observation, allowing a more flexible and patient-tailored admission policy.

## Introduction

Patients with hematological malignancies are known to be highly susceptible to infections, since the disease and/or therapy significantly weaken their immune system, leading to considerable infection-related mortality[1,2,3]. While the past decade has witnessed significant advances in treatment strategies for hematological cancers, prevention, and adequate management of infections still pose a major challenge. In a large retrospective study of more than 41,000 cancer patients admitted due to a suspected infection, mortality rates among those who were treated for leukemia, lymphoma, and myeloma were as high as 14.3%, 8.9%, and 8.2%, respectively[4]. Although for some patients with neutropenia developing during therapy for solid tumors, ambulatory treatment was reported to be safe[5], this could not be extrapolated to high-risk patients with hematological malignancies. Presently, there are no clear guidelines for the identification of hemato-oncological patients who could be treated in the ambulatory setting during post-chemotherapy neutropenia and who should be hospitalized either in a general internal medicine ward (GW), or in a hematological facility. In reality, the shortage of dedicated beds and economical restrictions provide an incentive for minimizing hospital stay. Obviously, patients with active life-threatening infections or other similar conditions should be admitted to hospital; however, many centers are re-evaluating their current practice in an attempt to reduce the number of "non-essential" hospitalizations. Commonly, in high-risk situations (e.g., induction therapy for acute leukemia), physicians choose to keep patients in the hospital for observation after completion of chemotherapy until the recovery of white blood cell (WBC) counts to ensure that immediate measures are taken in case of infection development. However, due to limited bed availability in Hematology Wards (HW), some patients are observed in GWs, or are discharged home shortly after chemotherapy completion. At multiple medical centers, discharged patients that developed fever or other signs of infection at home, receive emergency care at the Hematology Outpatient Clinic (HOutC) during morning hours on weekdays, whereas in the afternoon, at night, and during weekends, such patients are referred to the General Emergency Department (ED).

The well-established significance of rapid diagnostic measures and urgent initiation of antibiotic therapy at early signs of sepsis[6] and neutropenia[7] emphasizes the need for an emergency system dedicated to hemato-oncological patients. While such emergency facilities, termed Acute Oncology Service (AOS), are currently being promoted[8,9,10,11,12], no large-scale analysis of potential effects of this approach has been conducted yet, and its claimed benefits are supported by descriptive or qualitative evidence only. Additional emergency resources could also relieve acute medical units from the burden of managing complicated oncology patients[13,14].

Machine learning prediction models were shown to accurately predict patient outcome in several clinical situations, such as cardiac and general surgeries[15,16], ICU admissions, initiation of dialysis [17,18], etc. The majority of described infection predicting models focused on estimating the aggregated risk. A previous trial demonstrated that an artificial intelligence-based algorithm could successfully predict the development of sepsis in patients admitted to ICU[19]. Similarly, the present study was designated to develop a dynamic model for daily infection prediction. Additionally, we aimed to evaluate the rationale of keeping patients hospitalized for observation upon completion of chemotherapy and to compare the efficacy of a GW and an HW in such situations. The study intended to compare the outcome of hemato-oncological patients admitted and treated for infection in the ED/GW to that of patients whose infection was managed in dedicated facilities (HOutC and/or HW).

While scarce studies did develop models predicting daily infection risks, they were designed for a completely different clinical setting[20,21].

To the best of our knowledge, the present study is the first to quantitatively analyze the benefits of hematological emergency services and the impact of observational policy on infection and mortality hazard in patients with hematological malignancies.

## Methodology

The study was approved by the Institutional Review Board (Approval #066-14RMB), and was conducted in accordance with the Declaration of Helsinki. A waiver of patient informed consent was granted as it was a retrospective chart review study.

### Study design and setting

Rambam Health Care Campus is a tertiary hospital, providing advanced treatment for a population of about 2 million people. This retrospective cohort study encompassed all hematological patients who visited Rambam between 01/2011 and 10/2015. For these patients, electronic medical records (EMR) were reviewed starting from their first visit to the hospital (even before 2011) till 10/2015. Data obtained included patient demographics, ICD-9 codes, patient flow information throughout each visit, lab results, vital signs, administered medications [antibiotics, chemotherapy (CTX) drugs (S1 Table)], and major treatments [radiation therapy (RT), hematopoietic stem cell transplantation (HSCT), etc.]. While data were anonymized, different hospital visits of the same patient could be linked, using unique patient and visit numbers. If no or more than one ICD-9 code was recorded, a hematology physician accessed full patient records to determine the cancer diagnosis relevant to each visit.

### Model development

The current study included two stages. At the first stage, infection and mortality risk models for patients treated for hematological malignancies were built, taking into consideration patient clinical and laboratory parameters along with admission wards. These models identified the admission ward as a significant factor for infection and mortality risks. Of several prediction methods, we found the probit models to be the most accurate and allowing for daily evaluation of patient risk throughout the entire treatment cycle. The relative importance of an HW and a GW was estimated, using the number-needed-to-treat measurements to quantify the life-saving potential of the dedicated facilities. At the second stage, we built a prediction model for infection and mortality risks for a given patient if treated in a dedicated or general ward.

### Patient population and treatment definitions

During the study period, 8,786 patients visited the Adult HW and HOutC. Out of these, 2,330 had malignant hematological diseases and received treatment at the Rambam hospital. The evaluable patients were categorized into the following four groups. The Acute Leukemia (AL) group included patients with all subcategories of acute myeloid leukemia (AML), as well as acute lymphoblastic leukemia, and aplastic anemia. The Chronic Leukemia (CL) group included patients diagnosed with chronic myeloid leukemia, chronic myelomonocytic leukemia, chronic lymphocytic leukemia, high- and low-risk myelodysplastic syndrome, and myeloproliferative neoplasms. Patients diagnosed with any kind of lymphoma were incorporated into the Lymphoma group (L), and patients with multiple myeloma or any plasma cell discrasia formed the Multiple Myeloma group (MM). Only patients diagnosed with one (or more) of these diseases were included in the study.

A treatment protocol was defined as a combination of chemotherapy drugs prescribed to treat a specific disease at a predefined schedule. Each protocol regimen was administrated at repetitive cycles, which included active treatment and observation periods. The first day when CTX or RT was administered was considered the first day of an active treatment cycle. Each active cycle continued till the last day of CTX/RT administration. The observation period started on the first day of an interval of more than four consecutive days when a patient was off therapy. Consecutive days of CTX/RT treatment or treatment days that were separated by a weekend break were considered part of the same active cycle Consecutive treatments that were separated by at least four days of observation were considered separate cycles, even if they included identical drug combinations.

Among the 15,144 treatment cycles excluded from our analysis there were 523 cycles when an infection event occurred early during active therapy. We also excluded 1,092 cycles of therapy prescribed to patients within one year from hematopoietic stem cell transplantation (HSCT), as we could not differentiate between treatments prescribed for a post-transplant relapse and pre-planned maintenance therapy. In addition, 2,346 cycles were excluded due to the lack of important information, such as WBC counts.

We identified 4492 infection events, 176 of which were excluded due to missing data. Hence, the final models included 4,316 infection events that occurred following 11,183 treatment cycles.

Patient data were randomly divided into training (80%) and validation (20%) sets.

### Identification of infections in EMR

Since infections are not always accurately recorded on a daily basis, we developed an approach allowing for the identification of such events in EMRs. An infection was assumed to have occurred if at least one of the following took place: 1. a patient was admitted to hospital with an ICD-9 code indicating infection, 2. a patient had a fever higher than 38°C, or 3. a patient started antibiotic therapy that lasted for at least three consecutive days. The list of the antibiotic options is presented in S2 Table. Ciprofloxacin, fluconazole, and twice-weekly trimethoprim, commonly used for infection prophylaxis, were excluded from this list. We assumed an infection episode to last for 72 hours at least. If fever recurred or a new antibiotic was prescribed within 72 hours, we assumed these events to be related to the same infection episode. If fever higher than 38°C was documented after at least 72 hours of normal temperature, we considered this a new infection event. Application of this approach allowed for automatic identification of the events that were then verified and approved by physicians who double-checked random sample cases. Hospitalization of a patient without infection for more than 24 hours after an active treatment cycle was considered to occur merely for observation purposes.

### Outcomes and statistical analysis

The primary outcome evaluated in the study was the infection rate within 30 days after the end of each cycle of treatment. Specifically, we modeled the infection hazard rate on day *t* after the cycle completion, that is, the probability that a patient develops an infection on day *t*, given that he/she has not had an infection yet. The second study outcome was the death rate within 30 days after an infection event start. The data were randomly divided into two sets: the training set included 80% of the patients and the test set included 20% of the patients. Each of the outcomes was estimated using discrete-time survival (probit) models, allowing for the inclusion of time-varying variables, such as a change in the patient location within or outside the hospital, lab results, administered drugs, and vital signs [22,23,24]. Multi-collinearity tables of the features extracted from the data appear in Tables A-C in S1 File; Tables A-C and Fig B in S2 File, confirming that candidate variables are not highly correlated. Different models were compared using gradual (step-wise) addition of variables to attain the appropriate feature selection, and to better understand risk factor importance. Some of these models are presented herein and in Tables A-C in S1 File; Tables A-C and Fig B in S2 File. The main criterion for feature/model selection was the Bayesian information criterion (BIC). The BIC was selected because it penalized the number of features included in the model and therefore provided certain protection against over-fitting.

To establish the best possible prediction application, we compared the probit model to the following statistical methods: Logistic regression (Logit), Random forest, and Regression tree. Logit and Probit models have the advantage of interpretability, as it is relatively easy to understand the influence magnitude of each risk factor included in the model. Random forest was selected since it is known as one of the best machine learning methods for structured data classification problems[25]. Model prediction accuracy was evaluated using the test data based on the AUC measurements. All statistical analyses and figures were generated using the statistical programming environment R (the general GLM function was used for the statistical models, ggplot package was used for figure preparation) or MATLAB (version 9.5.0 and the statistical toolbox version 11.4).

## Results

### Patient characteristics

**Fig 1:**
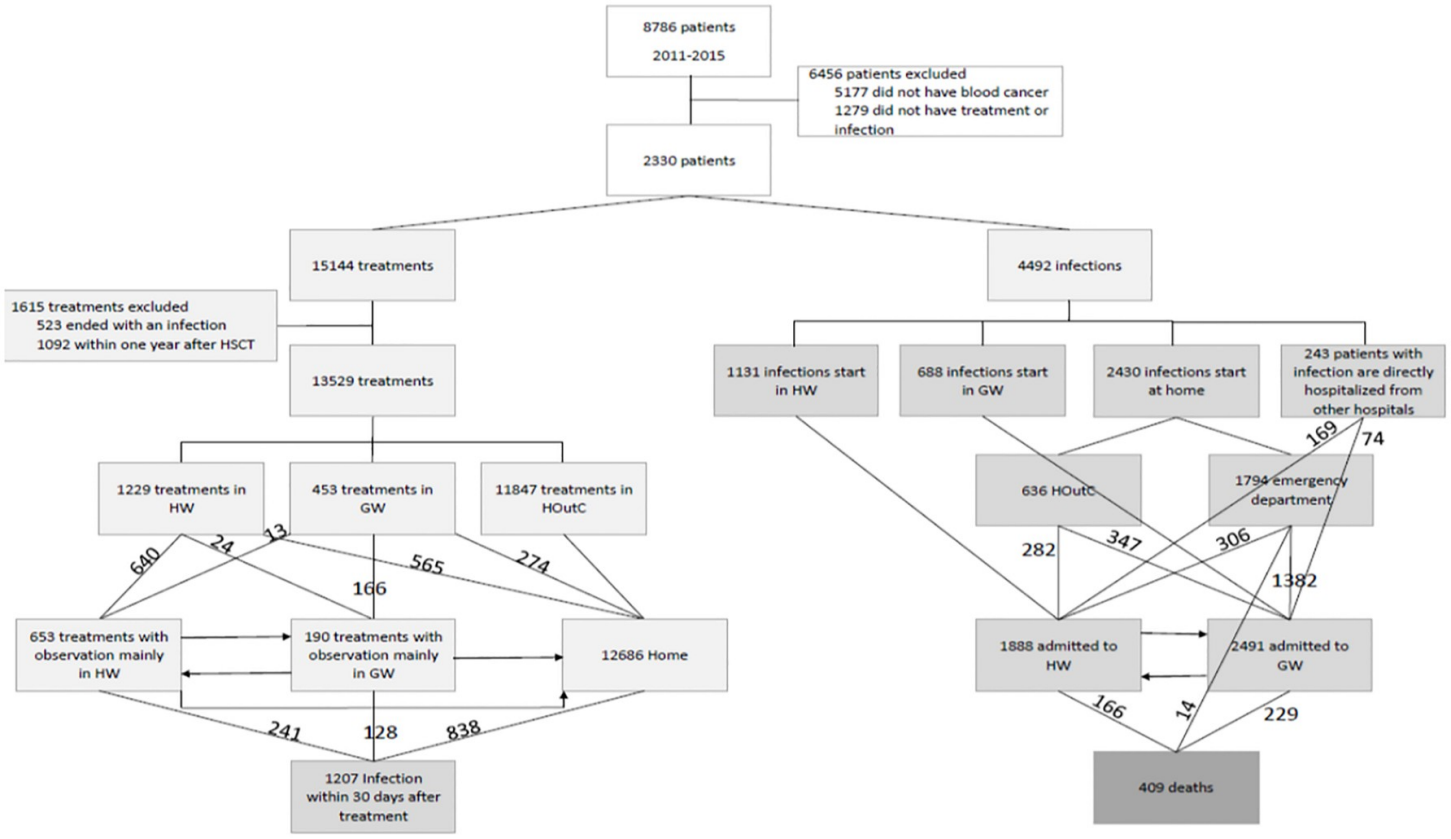
The study cohort flowchart. Left-hand branch: Patient location during and following administration of each chemotherapy cycle. Right-hand branch: Patient location following the diagnosis of an infection event.

Table 1 summarizes patient and treatment characteristics, while Fig 1 presents a flowchart of patient movements between hospital wards. Fifty five percent of all deaths recorded within the study period were infection-associated. The infection rate within 30 days upon chemotherapy completion was about 9% in the entire study population, but it was as high as 36.5% (ranging between 18.6 and 58.3% depending on the disease type) for the patients who received their treatment in the inpatient setting. The mean length of the observation period was four days. A total of 4,492 infection events occurred during the study period. About 9% of these events resulted in death, with this rate being comparable across the diseases. In 60% of patients, infection events started outside the hospital; 24% of these patients received emergency treatment in the HOutC, 67% were treated in the ED and 9% were directly admitted to the HW. Most patients in the latter group were suffering from serious conditions and were transferred from other local hospitals. The majority of patients (97%) with infection were hospitalized for anti-infection therapy, which on average lasted eight days. The choice of the inpatient facility where a specific patient was hospitalized [HW (30%) versus GW (70%)] depended on infection severity and bed availability.

**Table 1:** Patient and treatment characteristics.

	All N = 2,330	Acute Leukemia N = 558	Chronic Leukemia N = 219	Lymphoma N = 1,158	Multiple Myeloma N = 395	P-value^a^
Age, mean (95% CI)	55.5(54.9–56.2)	51.1(49.6–52.6)	60.0(58.2–61.8)	52.9(51.9–53.9)	59.8(58.6–61.1)	<0.001K
Number of females (%)	1,044 (44.8)	243 (43.5)	82 (37.4)	548 (47.3)	171 (43.3)	0.035C
Number of infection events, total per patient, mean (95% CI)	4,4922.13(2.03–2.23)	1,7773.51(3.28–3.75)	3661.93(1.63–2.23)	1,6841.59(1.46–1.71)	6651.89(1.69–2.09)	<0.001K
Number of chemotherapy cycles, total per patient, mean (95% CI)	13,5297.31(6.94–7.68)	1,3123.89(3.55–4.24)	7204.35(3.76–4.93)	7,1647.23(6.95–7.50)	4,33314.04(12.25–15.82)	<0.001K
Treatment length^b^1–5 days, No. (%)6-8 days, No. (%)9-10 days, No. (%)10+ days, No. (%)	11,636(86.01)1,037 (7.67)434 (3.21)422 (3.12)	798 (60.82)396 (30.18)16 (1.22)102 (7.77)	696 (96.67)20 (2.78)1 (0.14)3 (0.42)	6,755 (94.29)362 (5.05)19 (0.27)28 (0.39)	3,387 (78.17)259 (5.98)398 (9.19)289 (6.67)	
Number of drugs per treatment, mean (95% CI)	2.54(2.51–2.7)	1.54(1.49–1.58)	2.48(2.41–2.56)	3.36(3.32–3.40)	1.49(1.47–1.52)	<0.001K
Treatments with only newer-generation drugs, No. (%)Radiation treatments, No. (%)	1,536(11.35) 89 (0.66)	81 (6.17) 11 (0.84)	72(10) 4 (0.55)	1,316 (18.37) 43 (0.60)	67 (1.55) 31 (0.72)	<0.001C 0.716C
Antibiotics administered, No. (%):Anti-bacterial Anti-fungal Anti-viral No antibiotics	3,701 (82.39)258 (5.74)275 (6.12)346 (7.7)	1,426 (80.25)201 (11.31)102 (5.74)83 (4.67)	300 (81.97)8 (2.19)22 (6.01)43 (11.75)	1,390 (82.54)42 (2.49)100 (5.94)178 (10.57)	585 (87.97)7 (1.05)51 (7.67)42 (6.32)	<0.001C<0.001C0.344C<0.001C
Length of an infection event, average number of days (95% CI)	8.08(7.80–8.36)	10.90(10.33–11.48)	6.67(5.91–7.43)	6.14(5.83–6.44)	6.24(5.78–6.70)	<0.001K
Proportion of days in HW	40.96%	58.40%	21.21%	27.28%	39.88%	<0.001K
Proportion of days in GW	39.56%	27.28%	54.36%	49.17%	39.94%	<0.001K

^a^ P-value represents comparisons across diseases for each variable. For continuous variables, p-values were obtained from a Kruskal-Wallis [k] rank sum test, since equal variances or normality assumptions were often violated. For categorical variables, a Pearson’s Chi-squared [c] test was used.

^b^ The protocol length is the number of days between the first and the last day of chemotherapy, even if there is a break of ≤5 days between treatments.

### Infection survival and hazard rate

**Fig 2:**
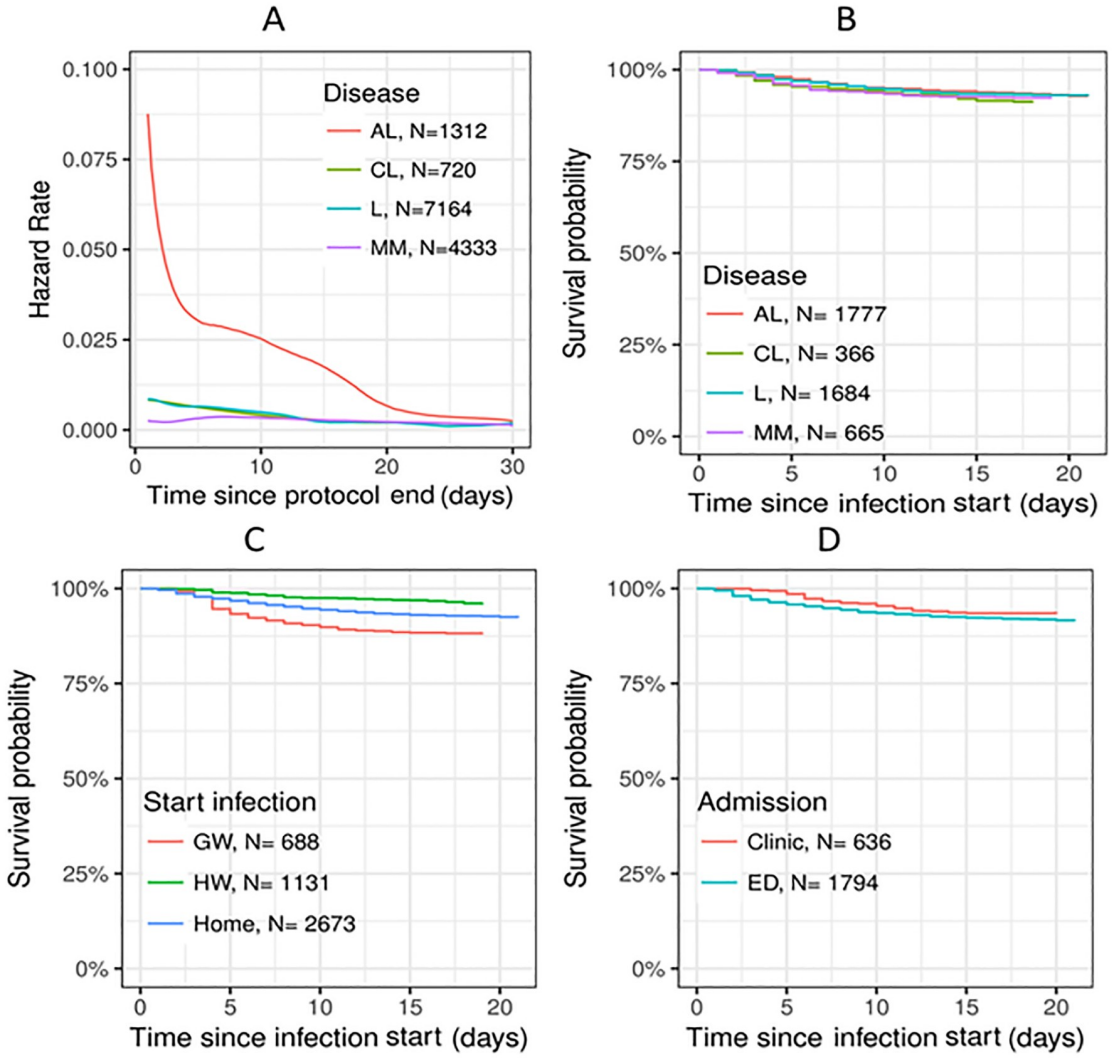
Infection hazard rate and survival after infection. Infection hazard during the first 30 days post-chemotherapy according to: A. The underlying hematological disease. Survival during the first 21 days following infection event according to: B. The underlying hematological disease. C. The setting where the infection event was diagnosed. D. The ward where emergency care was provided.

As shown in Fig 2, panel A, infection probability was highest on the first 2 to 3 days upon chemotherapy completion. As expected, the infection hazard was higher for AL patients and for treatments lasting six to eight days, such as induction for AML. Likewise, the protocols requiring hospitalization were associated with increased infection hazards. Data on post-infection survival are presented in Fig 2, panels B-D. The post-infection survival rate appeared to be related to the patient location (home, GW, or HW) at the time of infection event onset, and the mode of hospital admission (HOutC, ED, or direct hospitalization). Surprisingly, the survival rate of patients with infection was not related to the underlying hematological disease.

### Model 1—Infection hazard rate after chemotherapy completion

Results for the discrete-time probit model, predicting infection hazard for each of the first 30 days after every protocol cycle are presented in Table 2. The final model included the following explanatory factors: the protocol treatment length, the underlying disease, WBC counts on the last day of chemotherapy, the cycle number, patient’s age, and location during treatment and observation period (the correlation matrix appears in Fig A in S1 File). Based on sensitivity analysis, we coded the WBC count variable as a dummy variable distinguishing between high (i.e., more than 1,000 cells/μL) and low counts (Tables D-E and Fig A in S1 File, Table D in S2 File and S1 Fig). Protocols were also categorized based on the neutropenia-induction risk; specifically, we included the dummy variable *TrDrugsNew* to distinguish between the protocols exclusively including newer generation non-cytotoxic drugs (e.g., bortezomib, azacitidine), on the one hand, and commonly used chemotherapies, on the other hand.

**Table 2:** Model 1—Infection hazard rate after chemotherapy completion (training dataset).

Model parameters	Model without patient location information		Model with patient location information	
	Coefficients	Standard Error	Coefficients	Standard Error
Intercept	-0.9692***	0.1219	-0.7949***	0.1254
Number of days after chemotherapy completion (Day)	-0.0952***	0.0262	-0.0674*	0.0270
Day^2^ (A quadratic term of Day)	0.0018	0.0010	0.0013	0.0011
Patient location on a particular day (baseline is HW)				
GW			0.2340**	0.0906
Home			-0.5765***	0.0578
Age	0.0043***	0.0008	0.0034***	0.0009
Protocol length, (active cycle days—baseline is 6–8 days)				
1–5 days (TrLength(1,5])	-0.3129***	0.0375	-0.3196***	0.0382
9–10 days (TrLength(8,10])	-0.6828***	0.1372	-0.6688***	0.1370
>10 days (TrLength(10,Inf])	-0.3037***	0.0815	-0.2998***	0.0819
Location of chemotherapy delivery (baseline is HW)				
GW (TrGW)	-0.1850**	0.0698	-0.2761***	0.0798
Clinic (TrClinic)	-0.5248***	0.0335	-0.3748***	0.0379
Number of administered drug groups (TrNBDrugs)	0.0296**	0.0113	0.0378**	0.0115
Treatment included only newer-generation (non-CTX) drugs (TrDrugsNew)	-0.3199***	0.0711	-0.3072***	0.0719
First/Second treatment cycle (CycleNB1or2)	0.1853***	0.0299	0.1546***	0.0306
Number of previous infection events (InfNB)	0.0643***	0.0050	0.0623***	0.0051
WBC count >1000 at the end of treatment (TrEndWBC(1000,Inf])	-1.2756***	0.1199	-1.1889***	0.1229
Underlying hematological disease (baseline is acute leukemia)				
Chronic leukemia	0.1137	0.1835	0.2616	0.1901
Lymphoma	0.0688	0.0920	0.1790	0.0945
Multiple myeloma	-0.1891	0.1133	-0.0044	0.1173
Interaction of Day and TrEndWBC(1000,Inf]	0.1700***	0.0283	0.1776***	0.0288
Interaction of Day^2^ and TrEndWBC(1000,Inf]	-0.0056***	0.0011	-0.0061***	0.0011
Interaction of Day and chronic leukemia	-0.0935**	0.0359	-0.1169**	0.0372
Interaction of Day^2^ and chronic leukemia	0.0035*	0.0014	0.0042**	0.0015
Interaction of Day and lymphoma	-0.0782***	0.0174	-0.0989***	0.0180
Interaction of Day^2^ and lymphoma	0.0032***	0.0007	0.0038***	0.0008
Interaction of Day and multiple myeloma	-0.0424	0.0236	-0.0736**	0.0242
Interaction of Day^2^ and multiple myeloma	0.0019	0.0010	0.0029**	0.0010
AIC	9073.61		8894.21	
BIC	9320.45		9160.79	
Log Likelihood	-4511.80		-4420.10	
Deviance	9023.61		8840.21	
N	143424		143424	

Coefficients with

*p<0.05;

**p<0.01;

***p<0.001

The model baseline was an AL patient who got the first or second protocol treatment at the HW and stayed there for observation. The baseline protocol was 6–8 active treatment day long, involved newer-generation drugs only, and the WBC count on the last day on active treatment was lower than 1000. In case the location of chemotherapy administration was changed during a treatment cycle, the location where the patient received the last treatment was defined as the treatment location. The mean length was calculated only for the protocols including observation.

Of the variables incorporated in the model, the underlying hematological disease, the WBC count at the end of active treatment and the current day after treatment completion (Day and Day^2^) were found to significantly interact with each other. Examples of the conducted sensitivity analysis appear in Tables C-E in S1 File (for the categorization of WBC and cycle number variables).

At the feature selection stage, other models were also evaluated. Table 2 includes an additional baseline model that did not consider patient location information, and took into account only data related to patient medical history, along with both current and past treatments. Our findings showed that the location parameters were essential and significantly improved the model accuracy.

**Fig 3:**
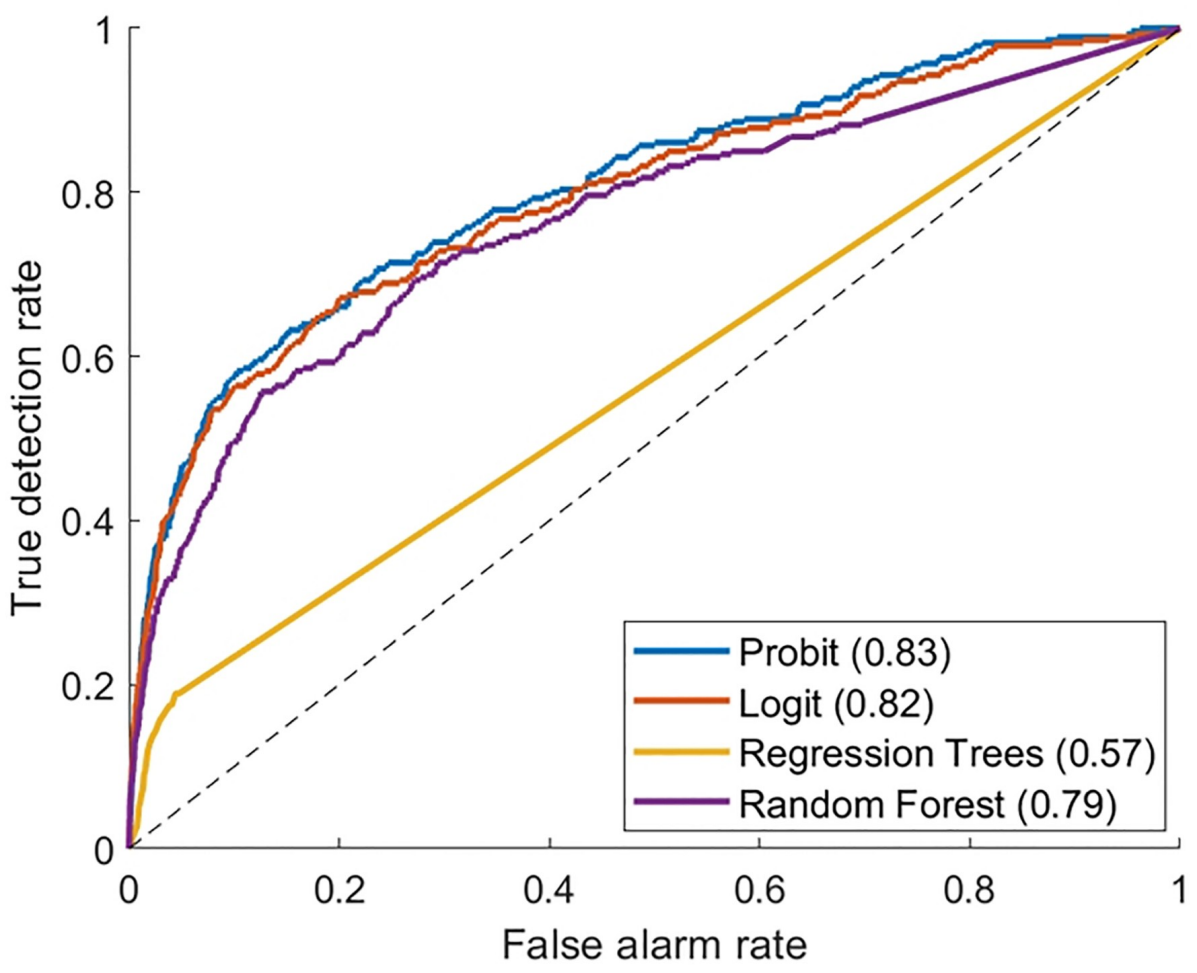
ROC curves for the infection models, using out-of-sample test data.

Superior infection-related results were demonstrated for the patients who stayed for observation in the HW than for those observed in the GW, where a higher infection hazard was revealed (p-value 0.009). The number needed to treat (NNT) for observation in the HW was 30.27, meaning that for every 30.27 days of observation in the HW one infection event was prevented. We compared the probit model to other statistical prediction methods, particularly to random forest, regression tree, and logit models. The ROC curves for these models are presented in Fig 3. While Probit, Logit and Random Forest exhibited similar results, the Probit appeared to be most accurate with an AUC of 0.83. Of note, the performance of the regression tree model was poor.

We have created a risk prediction tool (available at https://u0092023.shinyapps.io/InfectionHazards2/) that predicts infection development for each patient receiving any protocol treatment for any hematological malignancy.

### Model 2—Infection-related mortality hazard rate

Table 3 shows results for the discrete-time probit hazard model, estimating mortality hazard during the first 30 days after an infection event onset. Feature selection and sensitivity analysis were conducted in the same manner as for Model 1. The final model considered the impact of patient location (home, GW, or HW) at the time of infection onset and on each day during hospitalization as well as the mode of hospital admission (HOutC, ED, or direct hospitalization). Additionally, patient-related variables, such as age, WBC count at the beginning of an infection event, the number of previous infection events, and the number of administered treatment cycles, were taken into account.

**Table 3:** Model 2—Infection-related mortality hazard rate (training dataset).

Model parameters	Coefficient	Standard Error
Intercept	-3.6528***	0.1517
Location of the patient when the infection event started (baseline is home)		
HW	-0.3634***	0.0835
GW	0.0635	0.1089
Location of emergency treatment delivery for patients who developed infection at home (baseline is ED)		
Direct hospitalization (AdmDirectHosp)^§^	-0.3118*	0.1243
HOutC (AdmClinic)	-0.2077**	0.0771
First admission ward after emergency treatment (baseline is HW)		
GW (FirstHospGW)	-0.2248*	0.0971
Not admitted (i.e., discharged/died at ED/ HOutC) (NoHosp)	0.5498***	0.1660
Patient location on a particular day (baseline HW)		
GW	0.1787	0.0914
Home	-0.2817*	0.1306
ED	0.2886	0.1500
Number of days after an infection event started (InfectionDay)	0.0583***	0.0121
InfectionDay^2^ (A quadratic term of InfectionDay)	-0.0012**	0.0004
Age	0.0124***	0.0018
Cycle number (CycleNB)	0.0091**	0.0035
Infection developed after chemotherapy initiation (baseline is chemotherapy-unrelated infection)	0.2099**	0.0732
Number of previous infection events (InfNB)	0.0296***	0.0082
WBC count at the infection event start (baseline is [0,2000])		
(2000,15000]	-0.0267	0.0559
>15000	0.2610***	0.0710
Log likelihood	-1502.06	
Deviance	3004.12	
N	30033	

Coefficients with

*p<0.05;

**p<0.01;

***p<0.001

Model 2: The model baseline was an infection event starting at home for a patient with an initial WBC count lower than 2000, first treated at the ED and subsequently admitted to the HW.

^§^ Hospitalization was defined as direct when a patient was transferred from another hospital directly to the HW without passing through ED or HOutC.

**Fig 4:**
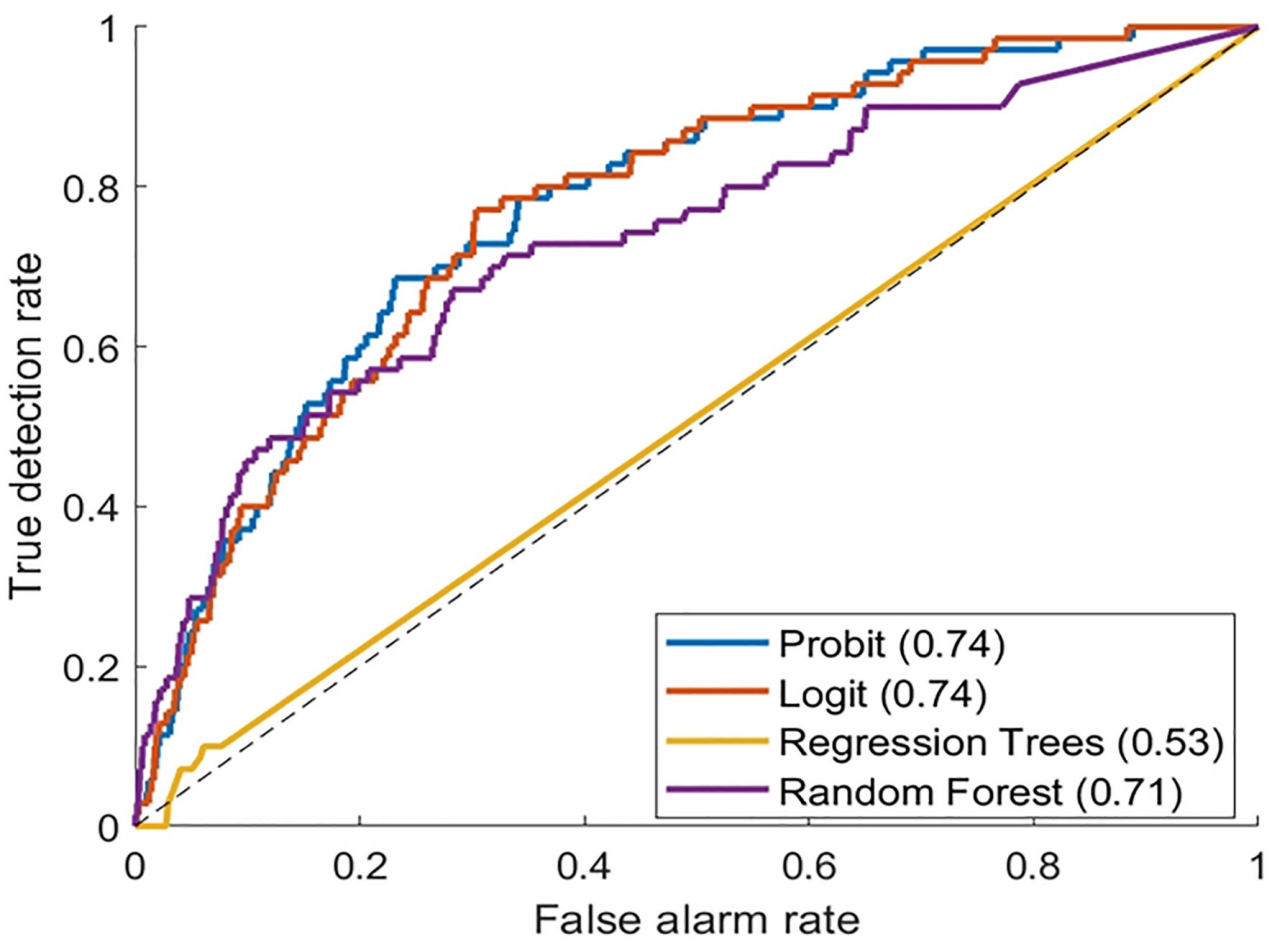
ROC curves for the mortality model, using test data (AUC values are indicated on the graph).

Patients presenting with infection were found to have a significantly superior survival rate if they received emergency treatment in the HOutC compared to those treated in the ED. The 21-day post-infection survival was 93.1% and 91.2%, respectively for these groups (Fig 2D). The NNT to save one life was 31.08 for the patients treated in the HOutC. Similar to model 1, this model was compared to other prediction methods. The ROC curves of several models are presented in Fig 4. Again, the probit model appeared to have the highest prediction power, with an AUC of 0.74 (results of Logit and Random Forest appear to be close). The final probit model was used to create a tool that predicts daily mortality risk based on individual patient data (available at https://u0092023.shinyapps.io/Mortality/).

### Time between the infection onset and the antibiotic therapy start

To elucidate why dedicated emergency units appeared to be more effective in treating infections in hematology patients we examined the time interval between the onset of an infection episode and the beginning of antibiotic therapy. Unfortunately, the database on hospitalized patients did not contain information regarding the exact time point of the first symptoms of infection. Fever measurement is an unreliable marker in such cases, since in this high-risk population physicians may diagnose an infection event based on minor symptoms or laboratory/imaging results, even when the patient is afebrile.

**Fig 5:**
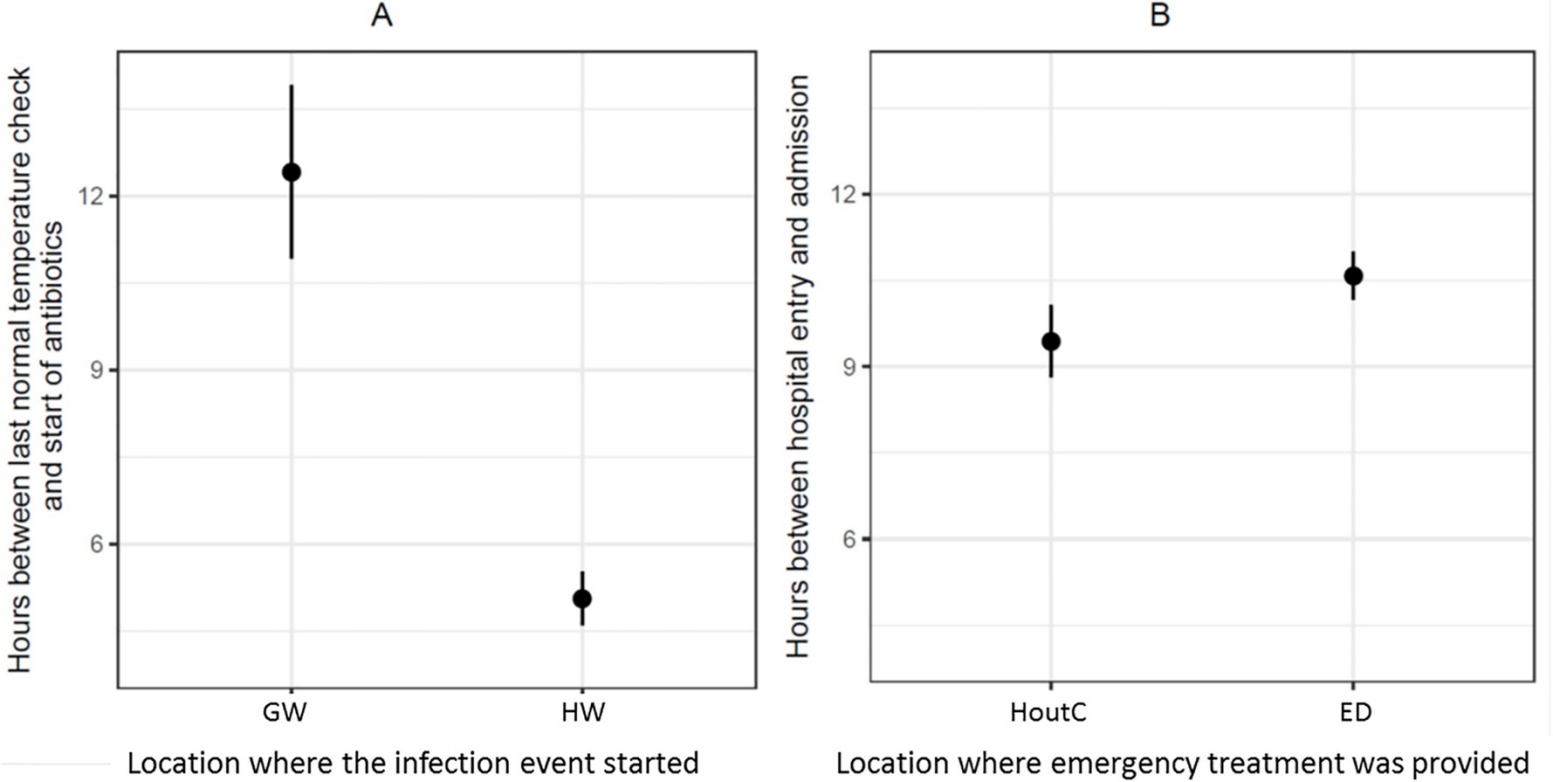
Estimated time lags in anti-infectious therapy initiation. Panel A shows the HW-GW differences in the estimated time lag between first signs of infection and antibiotic initiation, based on the time period between the last normal temperature record and the start of the antibiotic treatment. Panel B shows general ED-HOutC differences in the estimated time lag between patient arrival in hospital and completion of emergency evaluation and initiation of antibiotic therapy, based on the time of patient admission.

Therefore, for hospitalized patients, the upper limit of the symptoms-to-treatment time lag was approximated as the period between the last routine temperature reading below 38°C and the start of the antibiotic treatment (Fig 5A). For the patients whose infection event started at home, the time lag between the first symptoms of infection and arrival in hospital is unknown. Hence, the lower limit of the symptoms-to-treatment time lag for this patient group was the ED length-of-stay (time from admission till hospitalization) (Fig 5B). Time from admission through the general ED was associated with significant delays in antibiotic administration compared to admission through the HOutC (S2 Fig).

## Discussion

Among multiple toxicities and risks associated with intensive chemotherapy for hematological malignancies, life-threatening infection events are extremely common. This justifies the definition of patients suffering from these diseases as a population at risk that requires special considerations. The present study has demonstrated that patients treated for malignant hematological conditions have a better outcome when they are managed in specialized units. This is true both of observation upon chemotherapy completion and emergency treatment if an infection event occurs after discharge from hospital. Due to the shortage of such dedicated facilities, the identification of the patients who would benefit most from management in these wards is critical.

We have chosen the probit models as they provide more accurate results in predicting the risk of rare events, such as death. Since the interpretation of the coefficients is not intuitive [26], we have developed visual tools showing predictive values for infection hazard after treatment or mortality hazard following infection, which can be accessed online. These tools may be used for daily risk prediction for any patient, based on his/her demographics, specific disease, therapy administered, and location within the hospital. This approach can also help in assessing the dynamics of risk development on each particular day of observation and treatment periods.

Our models suggest that keeping high-risk patients in the HW for post-chemotherapy observation is effective for infection prevention and that the mortality hazard following an infection event is likely to be lower if emergency therapy is given in the HOutC compared to the general ED and even the GW. We are aware of the challenge of providing an understandable rationale for algorithm predictions[27] and have looked for potential explanations for our findings. Differences in infection hazard and related mortality rates cannot be attributed to variations in infection prevention or monitoring, since all units and departments at Rambam apply the same infection therapy strategy. Notably, despite the hospital admission policy advocating referral of patients with a high infection risk (e.g., acute leukemia) to the HW, the infection hazard in the HW is lower than in the GW. In all likelihood, a more aware staff, a better nurse-to-patient ratio resulting in higher nurse availability, and a faster response to symptoms or abnormal signs, may explain the reduction in infection or mortality rates in the dedicated units. We have demonstrated that a lag in response to high fever in the GW compared to the HW does exist, as reflected by longer intervals between the last normal temperature record and antibiotic therapy initiation. Manpower shortage and lack of awareness of the fragility of hematological patients may account for inferior outcomes of such patients, if observed in the GW.

The estimated contribution of a specific ward (HW/GW) where a patient is hospitalized upon receipt of emergency treatment for infection may be biased by hospital admission policy. At Rambam, hematological patients with severe infection are more likely to be admitted to the HW and as a result, the mortality hazard among patients first hospitalized in the GW is significantly lower (p-value 0.0206). However, for patients staying in the GW on subsequent days the mortality hazard is higher than for those hospitalized in the HW (p-value 0.0505). Remarkably, the underlying hematological disease is not found to be a significant risk factor for infection-related mortality. Yet, the mortality hazard appears to be significantly higher for heavily pre-treated patients, those who had many infection events in the past, or are of older age.

The current study has analyzed data derived from a single center. While this is a limitation, the homogeneity of the institutional infection prevention and treatment scheme reduces the impact of potentially confounding factors. Obviously, centers around the world differ in their patient population and the quality of care provided by their general wards. Our approach can be readily applied to other institutions with different standards of operation and EMR systems. Using this approach, patients at a high risk of life-threatening infection who will benefit from treatment in a dedicated facility can be identified and characterized in each particular center. Hence, the current study presents an efficient tool for identification and quantification of the patients requiring observation and treatment in specialized wards.

The decision of whether a patient should stay at hospital for observation is currently made upon completion of a chemotherapy course. The most frequently used criterion is the WBC count. This reflects myopic decision making. Once a patient is discharged, he/she will only return to hospital in case of an infection event.

Since our model provides temporal evaluation of patient risks, it may enable physicians to move from myopic (one-point) to a continuous dynamic decision making policy. With our tool, physicians can compare risks at different time points, which may result in a more flexible patient management policy. Importantly, the present study has demonstrated that while in some patients the infection risk is monotone decreasing, in others, the pattern appears to be increasing-decreasing. Our proposed calculator is capable of predicting the exact time point during post-chemotherapy observation when the infection risk in patients from the former group decreases to a level allowing their discharge from hospital. For the latter group that is usually discharged immediately after chemotherapy, one can calculate the exact date when the infection risk increases to a level justifying patient hospitalization. We are currently investigating the ways to identify optimal policies that minimize the expected combined infection and mortality risks, taking into account both patient point-of-view and capacity constraints. This is a very complicated optimization problem that is beyond the scope of the current paper.

As to the specialized emergency services, to the best of our knowledge, the present study is the first to provide quantitative support for the availability of such facilities for hematological patients. This is not trivial. For example, the queuing theory suggests that a larger ED may be more flexible and efficient in providing care to severely ill patients (since they are prioritized over regular ones). On the other hand, a small ED may provide a “concierge” treatment with faster reach of the required specialist. Our hospital actually chooses a mid-way solution, i.e., providing specialized ED services in the HOutC.

Our findings may have implications for future resource distribution in hospital development programs. HW beds are known to be expensive and cheaper substitutes are required. Combining the results of the two models developed in the current study suggest that establishing specialized ED or fast-track oncological emergency services could be more efficient in infection prevention and management than expanding the available GW.

In conclusion, our model for infection risk and associated mortality prediction among hemato-oncological patients has demonstrated that post-treatment observation in a specialized unit and dedicated emergency services may significantly improve patient outcome. Differences between dedicated hematological and general facilities are institution-specific, but the proposed model could be applied to any given EMR dataset, providing a reliable support tool for resource allocation decisions.

## Supporting information

S1 Table Overview of cytotoxic and non-cytotoxic anti-neoplastic drugs applied in the study population. (PDF)

S2 Table List of antibiotics commonly used for prophylaxis or active infection therapy in the study population. (PDF)

S1 File Infection after protocol completion: Model selection and sensitivity analysis. Table A. Comparison of models for infection after protocol completion analysis: Probit, logit and cloglog Table B. The location as an explanatory variable in the model for infection after protocol completion analysis Table C. Cycle number in the model for infection after protocol completion analysis Table D. The model for infection after protocol completion analysis with different WBC grouping Table E. White blood cell counts (mean or last) in the model for infection after protocol completion analysis Table F. Degrees of freedom (DF) of the predictor variables for the model of infection after protocol completion analysis Fig A. Multicollinearity checks and variance inflation factors (VIFs) for the model of infection after protocol completion analysis.
(PDF)

S2 File Mortality after infection: Model selection and sensitivity analysis. Table A. Comparison of models for mortality after infection analysis: Probit, logit, and cloglog Table B. The location as an explanatory variable in the model for mortality after infection analysis Table C. Cycle number in the model for mortality after infection analysis Table D. White blood cell counts in the model for mortality after infection analysis Table E. Type of infection in the model for mortality after infection analysis Table F. Time until antibiotic administration in the model of mortality after infection analysis Table G. Degrees of freedom (DF) of the predictor variables for the model of mortality after infection analysis Fig A. Time until antibiotic administration as a function of the location where the infection started and outcome (death) Fig B. Multicollinearity checks and variance inflation factors (VIFs).
(PDF)

S1 Fig Distribution of the WBC counts among the evaluated patients based on different factors (disease, protocol length, number of treatment cycles, hospitalization, infection occurrence, and patient age). (PDF)

S2 Fig Hospitalization delay by entry gate to the hospital (ED vs. HOutC) and the hour of admission. (PDF)
