# Supplementary material for: The role of specialized hospital units in infection and mortality risk reduction among patients with hematological cancers
Source: PLoS One. 2019 Mar 20;14(3):e0211694. doi: 10.1371/journal.pone.0211694 (PMC6426175; doi:10.1371/journal.pone.0211694)
Supplement: S1 Table — (PDF) [file pone.0211694.s001.pdf]

S1 Table. Overview of cytotoxic and non-cytotoxic anti-neoplastic drugs applied in the study population

Cytotoxic drugs			
1	Actinomycin	24	Gemzar, Gemcitabine
2	Alimta, Pemetrexed	25	Irinotecan, Topotecan, Hycamtin
3	Amsacrine	26	Leustatin, Cladrivine
4	Anti-human T-Lymphocyte globullin, Fresenius, Velcade, Bortezomib	27	Idarubicin, Daunorubicine, Cerubidin, Doxorubicin, Epirubicin, Farmorubicina, Motixantron, Nnovantrone
5	Asparginase, Kidrolase	28	Mabcampath
6	Avastin, Bevacizumab	29	Melphalan
7	Azacitidine, Vidaza	30	Methotrexate, Abitrexate
8	Bendamustin, Bleomycin	31	Methylmustine, nitr. Mustard Mustagen
9	Bleomycin	32	Mitomycin
10	Busulfex, Treosulfan	33	Mylotarg, Gemtuzumab Osogamicin
11	Carboplatin	34	Navelbine, Vinorelbine
12	Carfilzomib	35	Nelarabine, Atriance
13	Carmustine	36	Oxalipatin, Eloxatine
14	Cisplatin, Abiplatin	37	Procarbazine, Natulan
15	Clofarabine, Evoltra	38	Romidepsin istodax
16	Cyclophosphamide, Cytosphan, Ifosfamid, Ifoxan, Holoxan	39	Vinblastin, Velbe (blastovin), Vincristine, Oncovin, Cristovin
17	Cytarabine, Cytosar	40	Taxol, Paclitaxel, Taxotere, Docetaxel
18	Dacarbazine, Deticene	41	Telbivudine, Sebivo
19	Dacogen, Decitabine	42	Thiotepa
20	Difolta	43	Torisel, Temsirolimus
21	Etoposid, Vepesid	44	Uromitexan, Mesna
22	Fludarabine, Fludara	45	Vectibix, Panitumumumab
23	Fluorouracil	46	Yondelis,Trabectedin
**Non-cytotoxic drugs**			
47	Aredia, Pamidronate, Zomera, Zoledronic	54	Keytruda
48	Arsenic Trioxide	55	Obinutuzumab (Gazyva)

49	Brentuximab Vedotin	56	Octreotide, Sandostatin
50	Cetuximab, Erbitux	57	Opdivo
51	Eculizumab, Soliris	58	Ranibizumab (Lucentis)
52	Herceptin	59	Rituximad, Mabthera
53	Interferon alfa2B	60	Tretinoin (Vesanoid, ATRA)
61	Tacrolimus, Prograf, Ciclosporin, Sandimmune, Mycophenolate (Cellcept)		
