# Supplementary material for: The role of specialized hospital units in infection and mortality risk reduction among patients with hematological cancers
Source: PLoS One. 2019 Mar 20;14(3):e0211694. doi: 10.1371/journal.pone.0211694 (PMC6426175; doi:10.1371/journal.pone.0211694)
Supplement: S2 Table — (PDF) [file pone.0211694.s002.pdf]

S2 Table List of antibiotics commonly used for prophylaxis or active infection therapy in the study population

**Antibiotics commonly used for infection prophylaxis**
Ciprogis, Ciproxin, Fluconazole, Garamycin, Trican
**Antibiotics used for the management of active infections**
<u>Bacterial:</u><br>Amikacin, Ampicillin, Augmentin, Azactam, Azenil, Cefamezin, Ceforal, Ceftazidime, Chloramphenicol , Clindamycin, Daptomycin, Doxylin, Ertapenem, Erythrocin, Erythromycin, Flagyl, Fortum, Gentamicin, Linezolid, Imipenem, Klacid, Levofloxacin, Meronem, Meropenem, Metronidazole, Moxypen, Orbenil, Resprim, *Resprim forte*, Rifampin, Rocephin, Rulid, Septrin, Tavanic, Tazocin, Teicoplanin, Tygacil, Unasyn, Vancomycin, Zeto , Zinnat<br><u>Fungal:</u><br>Ambisome, Amphotericin, Caspofungin, Eraxis, Noxafil, Vfend, Voriconazole<br><u>Viral:</u><br>Cidofovir, Foscavir, Gancyclovir, Ribavirin, Tamiflu, Valganciclovir, *Zovirax*
