# Supplementary material for: The role of specialized hospital units in infection and mortality risk reduction among patients with hematological cancers
Source: PLoS One. 2019 Mar 20;14(3):e0211694. doi: 10.1371/journal.pone.0211694 (PMC6426175; doi:10.1371/journal.pone.0211694)
Supplement: S1 File — Table A. Comparison of models for infection after protocol completion analysis: Probit, logit and cloglog Table B. The location as an explanatory variable in the model for infection after protocol completion analysis Table C. Cycle number in the model for infection after protocol completion analysis Table D. The model for infection after protocol completion analysis with different WBC grouping Table E. White blood cell counts (mean or last) in the model for infection after protocol completion analysis Table F. Degrees of freedom (DF) of the predictor variables for the model of infection after protocol completion analysis Fig A. Multicollinearity checks and variance inflation factors (VIFs) for the model of infection after protocol completion analysis. (PDF) [file pone.0211694.s003.pdf]

## S3 File. Infection after protocol completion: model selection and sensitivity analysis

All models were chosen out of numerous others that were tried and rejected because of inferior BIC-values. Here we show these models, together with BIC values. We include an analysis of correlations between explanatory variables.

### Table A. Comparison of models for infection after protocol completion analysis: Probit, logit and cloglog

The final model is a probit model. The table below compares this model (on the left) with a logit or cloglog model. As the probit model has the lowest BIC value, it has been chosen.

| | Probit | Logit | Complementary log-log |
|---|---|---|---|
| (Intercept) | -.7949 (.1254)*** | -1.4842 (.2675)*** | -1.7228 (.2469)*** |
| DayNB | -.0674 (.0270)* | -.1189 (.0585)* | -.0991 (.0542) |
| DayNB2 | .0013 (.0011) | .0021 (.0024) | .0016 (.0023) |
| LocStartDayGW | .2340 (.0906)** | .4721 (.1934)* | .3990 (.1824)* |
| LocStartDayHome | -.5765 (.0578)*** | -1.2458 (.1295)*** | -1.1794 (.1256)*** |
| Age | .0034 (.0008)*** | .0076 (.0022)*** | .0072 (.0022)*** |
| CycleNB1or2TRUE | .1546 (.0306)*** | .3894 (.0779)*** | .3702 (.0763)*** |
| InfNB | .0623 (.0051)*** | .1436 (.0123)*** | .1356 (.0120)*** |
| TrLength05TRUE | -.3196 (.0382)*** | -.7419 (.0919)*** | -.7010 (.0896)*** |
| TrLength810TRUE | -.6688 (.1370)*** | -1.8105 (.4224)*** | -1.7693 (.4211)*** |
| TrLength10∞TRUE | -.2998 (.0819)*** | -.6453 (.2005)** | -.5866 (.1937)** |
| TrLocGW | -.2761 (.0798)*** | -.5912 (.1915)** | -.5255 (.1853)** |
| TrLocClinic | -.3748 (.0379)*** | -.9646 (.0974)*** | -.9600 (.0966)*** |
| TrNBDrugGroups | .0378 (.0115)** | .0929 (.0299)** | .0895 (.0294)** |
| newdrugsTRUE | -.3072 (.0719)*** | -1.1027 (.2239)*** | -1.1116 (.2230)*** |
| TrEndWBC(1000,∞] | -1.1889 (.1229)*** | -2.5109 (.2599)*** | -2.2982 (.2429)*** |
| ChronicLeukemia | .2616 (.1901) | .8731 (.5023) | .8585 (.4961) |
| Lymphoma | .1790 (.0945) | .5327 (.2296)* | .5265 (.2247)* |
| MultipleMyeloma | -.0044 (.1173) | -.0543 (.3147) | -.0806 (.3113) |
| DayNB:TrEndWBC(1000,∞] | .1776 (.0288)*** | .4108 (.0646)*** | .3814 (.0612)*** |
| DayNB2:TrEndWBC(1000,∞] | -.0061 (.0011)*** | -.0150 (.0027)*** | -.0141 (.0026)*** |
| DayNB:ChronicLeukemia | -.1169 (.0372)** | -.3331 (.1042)** | -.3267 (.1033)** |
| DayNB2:ChronicLeukemia | .0042 (.0015)** | .0115 (.0043)** | .0112 (.0043)** |
| DayNB:Lymphoma | -.0989 (.0180)*** | -.2559 (.0452)*** | -.2500 (.0444)*** |
| DayNB2:Lymphoma | .0038 (.0008)*** | .0096 (.0020)*** | .0093 (.0019)*** |
| DayNB:MultipleMyeloma | -.0736 (.0242)** | -.1763 (.0671)** | -.1683 (.0665)* |
| DayNB2:MultipleMyeloma | .0029 (.0010)** | .0065 (.0030)* | .0062 (.0030)* |
| AIC | 8894.2057 | 8941.2683 | 8956.0442 |
| BIC | 9160.7919 | 9207.8544 | 9222.6303 |
| Log Likelihood | -4420.1029 | -4443.6341 | -4451.0221 |
| Deviance | 8840.2057 | 8887.2683 | 8902.0442 |
| Num. obs. | 143424 | 143424 | 143424 |
| Coefficients marked with * indicate p<0.05; ** indicate p<0.01; *** indicate p<0.001. | | | |
| | | | |

**Table B. The location as an explanatory variable in the model for infection after protocol completion analysis**

An important element in the chosen model is the location variable. This categorical variable is represented by the two dummy variables, *GW* and *Home*, which is one for the patient who is in the general ward or at home on a particular day. If both dummy variables are zero, the patient is in the hematology ward (HW). S3 Table B shows that a model with only location variables or only patient or treatment variables has higher BIC values, i.e., performs worse.

| | **Chosen model** | **Only location** | **No location** |
|---|---|---|---|
| Intercept | -.7949 (.1254)*** | -1.3763 (.0399)*** | -.9692 (.1219)*** |
| Day | -.0674 (.0270)* | | -.0952 (.0262)*** |
| Day2 | .0013 (.0011) | | .0018 (.0010) |
| GW | .2340 (.0906)** | -.1525 (.0751)* | |
| Home | -.5765 (.0578)*** | -1.2282 (.0421)*** | |
| Age | .0034 (.0008)*** | | .0043 (.0008)*** |
| CycleNB1or2 | .1546 (.0306)*** | | .1853 (.0299)*** |
| InfNB | .0623 (.0051)*** | | .0643 (.0050)*** |
| TrLength(0,5] | -.3196 (.0382)*** | | -.3129 (.0375)*** |
| TrLength(5,10] | -.6688 (.1370)*** | | -.6828 (.1372)*** |
| TrLength(10,Inf] | -.2998 (.0819)*** | | -.3037 (.0815)*** |
| TrGW | -.2761 (.0798)*** | | -.1850 (.0698)** |
| TrClinic | -.3748 (.0379)*** | | -.5248 (.0335)*** |
| TrNBDrugs | .0378 (.0115)** | | .0296 (.0113)** |
| TrDrugsOld | -.3072 (.0719)*** | | -.3199 (.0711)*** |
| TrEndWBC(1000,Inf] | -1.1889 (.1229)*** | | -1.2756 (.1199)*** |
| ChronicLeukemia | .2616 (.1901) | | .1137 (.1835) |
| Lymphoma | .1790 (.0945) | | .0688 (.0920) |
| MultipleMyeloma | -.0044 (.1173) | | -.1891 (.1133) |
| Day:TrEndWBC(1000,Inf] | .1776 (.0288)*** | | .1700 (.0283)*** |
| Day2:TrEndWBC(1000,Inf] | -.0061 (.0011)*** | | -.0056 (.0011)*** |
| Day:ChronicLeukemia | -.1169 (.0372)** | | -.0934 (.0359)** |
| Day2:ChronicLeukemia | .0042 (.0015)** | | .0035 (.0014)* |
| Day:Lymphoma | -.0989 (.0180)*** | | -.0782 (.0174)*** |
| Day2:Lymphoma | .0038 (.0008)*** | | .0032 (.0007)*** |
| Day:MultipleMyeloma | -.0736 (.0242)** | | -.0424 (.0236) |
| Day2:MultipleMyeloma | .0029 (.0010)** | | .0019 (.0010) |
| AIC | 8894.2057 | 9868.6702 | 9073.6061 |
| BIC | 9160.7919 | 9898.2908 | 9320.4451 |
| Log Likelihood | -4420.1029 | -4931.3351 | -4511.8031 |
| Deviance | 8840.2057 | 9862.6702 | 9023.6061 |
| Num. obs. | 143424 | 143424 | 143424 |
| Coefficients marked with * indicate p<0.05; ** indicate p<0.01; *** indicate p<0.001. | | | |

**Table C. Cycle number in the model for infection after protocol completion analysis**

The final model separates predictions for the first and second cycle. This model has a lower BIC value than a model with only the first or second cycle as a dummy variable (2nd and 3rd column in the table below) or one with the cycle number as a continuous variable (last columns in the table below).

| | Original model | CycleNB1 | CycleNB2 | Continuous CycleNB |
|---|---|---|---|---|
| (Intercept) | -.7949 (.1254)*** | -.7686 (.1249)*** | -.7038 (.1237)*** | -.6523 (.1238)*** |
| DayNB | -.0674 (.0270)* | -.0682 (.0270)* | -.0713 (.0269)** | -.0710 (.0270)** |
| DayNB2 | .0013 (.0011) | .0013 (.0011) | .0014 (.0011) | .0014 (.0011) |
| LocStartDayGW | .2340 (.0906)** | .2533 (.0909)** | .2239 (.0906)* | .2318 (.0908)* |
| LocStartDayHome | -.5765 (.0578)*** | -.5558 (.0584)*** | -.6041 (.0576)*** | -.5873 (.0575)*** |
| Age | .0034 (.0008)*** | .0034 (.0008)*** | .0035 (.0008)*** | .0036 (.0008)*** |
| CycleNB1or2TRUE | .1546 (.0306)*** | | | |
| InfNB | .0623 (.0051)*** | .0615 (.0051)*** | .0604 (.0051)*** | .0633 (.0051)*** |
| TrLength05TRUE | -.3196 (.0382)*** | -.3219 (.0382)*** | -.3309 (.0381)*** | -.3244 (.0381)*** |
| TrLength810TRUE | -.6688 (.1370)*** | -.6665 (.1375)*** | -.6567 (.1375)*** | -.7327 (.1377)*** |
| TrLength10∞TRUE | -.2998 (.0819)*** | -.3006 (.0819)*** | -.3073 (.0822)*** | -.3389 (.0818)*** |
| TrLocGW | -.2761 (.0798)*** | -.2956 (.0801)*** | -.2587 (.0800)** | -.2587 (.0802)** |
| TrLocClinic | -.3748 (.0379)*** | -.3805 (.0376)*** | -.3881 (.0376)*** | -.3671 (.0379)*** |
| TrNBDrugGroups | .0378 (.0115)** | .0382 (.0115)*** | .0415 (.0114)*** | .0311 (.0116)** |
| newdrugsTRUE | -.3072 (.0719)*** | -.3132 (.0716)*** | -.3117 (.0718)*** | -.3260 (.0718)*** |
| TrEndWBC(1000,∞] | -1.1889 (.1229)*** | -1.1898 (.1229)*** | -1.2051 (.1224)*** | -1.2100 (.1226)*** |
| ChronicLeukemia | .2616 (.1901) | .2464 (.1897) | .2635 (.1899) | .2568 (.1895) |
| Lymphoma | .1790 (.0945) | .1556 (.0942) | .1635 (.0940) | .1755 (.0939) |
| MultipleMyeloma | -.0044 (.1173) | -.0358 (.1169) | -.0289 (.1169) | .0678 (.1187) |
| DayNB:TrEndWBC(1000,∞] | .1776 (.0288)*** | .1775 (.0288)*** | .1807 (.0287)*** | .1795 (.0288)*** |
| DayNB2:TrEndWBC(1000,∞] | -.0061 (.0011)*** | -.0061 (.0011)*** | -.0062 (.0011)*** | -.0061 (.0011)*** |
| DayNB:ChronicLeukemia | -.1169 (.0372)** | -.1171 (.0370)** | -.1163 (.0371)** | -.1160 (.0371)** |
| DayNB2:ChronicLeukemia | .0042 (.0015)** | .0042 (.0015)** | .0041 (.0015)** | .0041 (.0015)** |
| DayNB:Lymphoma | -.0989 (.0180)*** | -.0979 (.0180)*** | -.0973 (.0179)*** | -.0973 (.0179)*** |
| DayNB2:Lymphoma | .0038 (.0008)*** | .0038 (.0008)*** | .0037 (.0007)*** | .0037 (.0007)*** |
| DayNB:MultipleMyeloma | -.0736 (.0242)** | -.0729 (.0242)** | -.0727 (.0241)** | -.0703 (.0243)** |
| DayNB2:MultipleMyeloma | .0029 (.0010)** | .0029 (.0010)** | .0028 (.0010)** | .0027 (.0010)* |
| CycleNB1TRUE | | .1835 (.0389)*** | | |
| CycleNB2TRUE | | | .0680 (.0383) | |
| CycleNB | | | | -.0097 (.0021)*** |
| AIC | 8894.2057 | 8897.6468 | 8916.0433 | 8895.9727 |
| BIC | 9160.7919 | 9164.2329 | 9182.6295 | 9167.5589 |
| Log Likelihood | -4420.1029 | -4421.8234 | -4431.0217 | -4418.4864 |
| Deviance | 8840.2057 | 8843.6468 | 8862.0433 | 8836.9727 |
| Num. obs. | 143424 | 143424 | 143424 | 143424 |
| Coefficients marked with * indicate p<0.05; ** indicate p<0.01; *** indicate p<0.001. | | | | |

**White blood cell counts at the end of protocol**

In the chosen model, patients that end protocol with a WBC below 1000 are predicted to have a significantly higher infection hazard rate. We also tried to make more categories (dummy variables) for the WBC, as suggested by physicians, but that did not result in lower BIC. The next table shows yet 4 models where WBC was included differently. The first one (mean WBC) has the mean WBC on each day as an explanatory variable. Since the WBC was not measured on each day after protocol (for instance when the patient is at home), we have substantially less observations (only 8431 days instead of 143424 in the chosen model). Instead of using the WBC at the end of treatment, we can also look at the last WBC that was measured, whether it is a couple of hours or several days prior. The second and third columns display this model. These models have slightly more observations and that means de BIC value cannot be directly compared to the chosen model. These models, however, largely show the same trends as the chosen model and we opted for the latter since it was simpler.

**Table D. The model for infection after protocol completion analysis with different WBC grouping**

| | **Chosen model** | **{0,1000,4000,∞}** | **{0,1000,15000, ∞}** | **{0,1000,4000, 15000, ∞}** |
|---|---|---|---|---|
| (Intercept) | -.7949 (.1254)*** | -.8466 (.1261)*** | -.7861 (.1254)*** | -.8441 (.1261)*** |
| DayNB | -.0674 (.0270)* | -.0657 (.0270)* | -.0676 (.0270)* | -.0655 (.0270)* |
| DayNB2 | .0013 (.0011) | .0012 (.0011) | .0013 (.0011) | .0012 (.0011) |
| LocStartDayGW | .2340 (.0906)** | .2387 (.0907)** | .2354 (.0906)** | .2399 (.0907)** |
| LocStartDayHome | -.5765 (.0578)*** | -.5719 (.0579)*** | -.5737 (.0578)*** | -.5684 (.0579)*** |
| Age | .0034 (.0008)*** | .0038 (.0009)*** | .0033 (.0009)*** | .0038 (.0009)*** |
| CycleNB1or2TRUE | .1546 (.0306)*** | .1569 (.0307)*** | .1481 (.0307)*** | .1498 (.0309)*** |
| InfNB | .0623 (.0051)*** | .0623 (.0051)*** | .0618 (.0051)*** | .0618 (.0051)*** |
| TrLength05TRUE | -.3196 (.0382)*** | -.2991 (.0386)*** | -.3212 (.0381)*** | -.2986 (.0386)*** |
| TrLength810TRUE | -.6688 (.1370)*** | -.6551 (.1376)*** | -.6662 (.1370)*** | -.6502 (.1376)*** |
| TrLength10∞TRUE | -.2998 (.0819)*** | -.2847 (.0820)*** | -.2967 (.0819)*** | -.2795 (.0821)*** |
| TrLocGW | -.2761 (.0798)*** | -.2684 (.0797)*** | -.2899 (.0798)*** | -.2818 (.0797)*** |
| TrLocClinic | -.3748 (.0379)*** | -.3785 (.0379)*** | -.3821 (.0379)*** | -.3868 (.0380)*** |
| TrNBDrugGroups | .0378 (.0115)** | .0376 (.0116)** | .0379 (.0115)*** | .0378 (.0115)** |
| newdrugsTRUE | -.3072 (.0719)*** | -.3014 (.0722)*** | -.3062 (.0720)*** | -.2975 (.0723)*** |
| TrEndWBC(1000,∞] | -1.1889 (.1229)*** | | | |
| ChronicLeukemia | .2616 (.1901) | .3645 (.1915) | .2029 (.1943) | .2838 (.1954) |
| Lymphoma | .1790 (.0945) | .2666 (.0967)** | .1823 (.0944) | .2819 (.0966)** |
| MultipleMyeloma | -.0044 (.1173) | .1231 (.1208) | .0148 (.1176) | .1672 (.1214) |
| DayNB:TrEndWBC(1000,∞] | .1776 (.0288)*** | | | |
| DayNB2:TrEndWBC(1000,∞] | -.0061 (.0011)*** | | | |
| DayNB:ChronicLeukemia | -.1169 (.0372)** | -.1295 (.0375)*** | -.1079 (.0379)** | -.1179 (.0382)** |
| DayNB2:ChronicLeukemia | .0042 (.0015)** | .0044 (.0015)** | .0037 (.0015)* | .0039 (.0015)* |
| DayNB:Lymphoma | -.0989 (.0180)*** | -.1093 (.0185)*** | -.0985 (.0180)*** | -.1106 (.0185)*** |
| DayNB2:Lymphoma | .0038 (.0008)*** | .0040 (.0008)*** | .0038 (.0008)*** | .0041 (.0008)*** |
| DayNB:MultipleMyeloma | -.0736 (.0242)** | -.0889 (.0248)*** | -.0759 (.0242)** | -.0944 (.0249)*** |
| DayNB2:MultipleMyeloma | .0029 (.0010)** | .0032 (.0011)** | .0030 (.0010)** | .0034 (.0011)** |
| TrEndWBC(1000,4000] | | -1.0365 (.1269)*** | | -1.0453 (.1269)*** |

| | | | | |
|---|---|---|---|---|
| TrEndWBC(4000,∞] | | -1.3891 (.1299)*** | | |
| DayNB:TrEndWBC(1000,4000] | | .1608 (.0300)*** | | .1615 (.0300)*** |
| DayNB:TrEndWBC(4000,∞] | | .2003 (.0299)*** | | |
| DayNB2:TrEndWBC(1000,4000] | | -.0058 (.0012)*** | | -.0058 (.0012)*** |
| DayNB2:TrEndWBC(4000, ∞] | | -.0066 (.0012)*** | | |
| TrEndWBC(1000,15000] | | | -1.2086 (.1234)*** | |
| TrEndWBC(15000, ∞] | | | -.9520 (.1803)*** | -1.0195 (.1805)*** |
| DayNB:TrEndWBC(1000,15000] | | | .1803 (.0289)*** | |
| DayNB:TrEndWBC(15000, ∞] | | | .1493 (.0384)*** | .1564 (.0385)*** |
| DayNB2:TrEndWBC(1000,15000] | | | -.0062 (.0011)*** | |
| DayNB2:TrEndWBC(15000, ∞] | | | -.0047 (.0015)** | -.0048 (.0015)** |
| TrEndWBC(4000,15000] | | | | -1.4473 (.1316)*** |
| DayNB:TrEndWBC(4000,15000] | | | | .2079 (.0301)*** |
| DayNB2:TrEndWBC(4000,15000] | | | | -.0069 (.0012)*** |
| AIC | 8894.2057 | 8870.6707 | 8889.4127 | 8861.0141 |
| BIC | 9160.7919 | 9166.8775 | 9185.6195 | 9186.8416 |
| Log Likelihood | -4420.1029 | -4405.3353 | -4414.7064 | -4397.5071 |
| Deviance | 8840.2057 | 8810.6707 | 8829.4127 | 8795.0141 |
| Num. obs. | 143424 | 143424 | 143424 | 143424 |

Coefficients marked with * indicate p<0.05; ** indicate p<0.01; *** indicate p<0.001.

**Table E. White blood cell counts (mean or last) in the model for infection after protocol completion analysis**

| | Mean WBC | Last WBC | LastWBC + interactions |
|---|---|---|---|
| (Intercept) | -.7947 (.1993)*** | -1.2369 (.1114)*** | -1.1566 (.1401)*** |
| DayNB | -.1063 (.0377)** | .0037 (.0135) | .0097 (.0249) |
| DayNB2 | .0034 (.0017)* | -.0008 (.0005) | -.0025 (.0012)* |
| LocStartDayGW | .0914 (.1114) | .2494 (.0873)** | .2623 (.0882)** |
| LocStartDayHome | .2926 (.0733)*** | -.5273 (.0589)*** | -.4976 (.0604)*** |
| Age | .0057 (.0013)*** | .0055 (.0008)*** | .0056 (.0009)*** |
| CycleNB1or2TRUE | .1071 (.0452)* | .1146 (.0297)*** | .1166 (.0300)*** |
| InfNB | .0558 (.0077)*** | | |
| TrLength05TRUE | -.0845 (.0557) | | |
| TrLength810TRUE | -.6733 (.1897)*** | | |
| TrLength10infTRUE | -.1761 (.1156) | | |
| TrLocGW | .0554 (.1166) | .0709 (.0681) | .0541 (.0680) |
| TrLocClinic | .1422 (.0589)* | -.1545 (.0405)*** | -.1770 (.0408)*** |
| TrNBDrugGroups | .0670 (.0167)*** | .0020 (.0115) | -.0018 (.0116) |
| newdrugsTRUE | -.0911 (.1160) | | |
| TrEndWBC(1000,Inf] | -.5589 (.1676)*** | | |
| ChronicLeukemia | .2590 (.2760) | .1351 (.1635) | .2987 (.1756) |
| Lymphoma | .3787 (.1294)** | .1071 (.0889) | .2764 (.1033)** |
| MultipleMyeloma | .5298 (.1732)** | -.0756 (.1083) | .1412 (.1251) |
| WBCMeanDayCat(100,1e+03] | -.2448 (.1275) | | |
| WBCMeanDayCat(1e+03,4e+03] | -1.1589 (.1341)*** | | |
| WBCMeanDayCat(4e+03,1.5e+04] | -1.2504 (.1358)*** | | |
| WBCMeanDayCat(1.5e+04,Inf] | -1.0745 (.1474)*** | | |
| DayNB:TrEndWBC(1000,Inf] | .1486 (.0421)*** | | |
| DayNB2:TrEndWBC(1000,Inf] | -.0055 (.0018)** | | |
| DayNB:ChronicLeukemia | -.0650 (.0528) | -.0289 (.0296) | -.0450 (.0312) |

| | | | |
|---|---|---|---|
| DayNB2:ChronicLeukemia | .0021 (.0020) | .0010 (.0011) | .0009 (.0011) |
| DayNB:Lymphoma | -.0666 (.0256)** | -.0162 (.0159) | -.0322 (.0179) |
| DayNB2:Lymphoma | .0025 (.0010)* | .0006 (.0006) | .0004 (.0007) |
| DayNB:MultipleMyeloma | -.1006 (.0346)** | .0075 (.0212) | -.0143 (.0234) |
| DayNB2:MultipleMyeloma | .0030 (.0014)* | -.0002 (.0008) | -.0002 (.0009) |
| WBCLastCat(500,1e+03] | | -.5231 (.0553)*** | -.5307 (.1627)** |
| WBCLastCat(1e+03,4e+03] | | -1.3080 (.0476)*** | -1.6739 (.1376)*** |
| WBCLastCat(4e+03,1.5e+04] | | -1.5625 (.0499)*** | -1.8803 (.1403)*** |
| WBCLastCat(1.5e+04,Inf] | | -1.0776 (.0615)*** | -1.2351 (.1682)*** |
| TrLength(5,8] | | .1613 (.0388)*** | .1468 (.0394)*** |
| TrLength(8,10] | | -.3983 (.1341)** | -.4000 (.1339)** |
| TrLength(10,Inf] | | .0852 (.0738) | .0655 (.0746) |
| TrDrugsOldGeneration | | .3359 (.0718)*** | .3517 (.0717)*** |
| DayNB:WBCLastCat(500,1e+03] | | | .0121 (.0366) |
| DayNB:WBCLastCat(1e+03,4e+03] | | | .0373 (.0285) |
| DayNB:WBCLastCat(4e+03,1.5e+04] | | | .0084 (.0285) |
| DayNB:WBCLastCat(1.5e+04,Inf] | | | -.0088 (.0333) |
| DayNB2:WBCLastCat(500,1e+03] | | | -.0006 (.0018) |
| DayNB2:WBCLastCat(1e+03,4e+03] | | | .0007 (.0013) |
| DayNB2:WBCLastCat(4e+03,1.5e+04] | | | .0024 (.0013) |
| DayNB2:WBCLastCat(1.5e+04,Inf] | | | .0025 (.0015) |
| AIC | 4703.4208 | 9419.0050 | 9344.6349 |
| BIC | 4921.6506 | 9687.3679 | 9692.5128 |
| Log Likelihood | -2320.7104 | -4682.5025 | -4637.3175 |
| Deviance | 4641.4208 | 9365.0050 | 9274.6349 |
| Num. obs. | 8431 | 153180 | 153180 |

Coefficients marked with * indicate p<0.05; ** indicate p<0.01; *** indicate p<0.001.

**Fig A. Multicollinearity checks and variance inflation factors (VIFs) for the model of infection after protocol completion analysis**

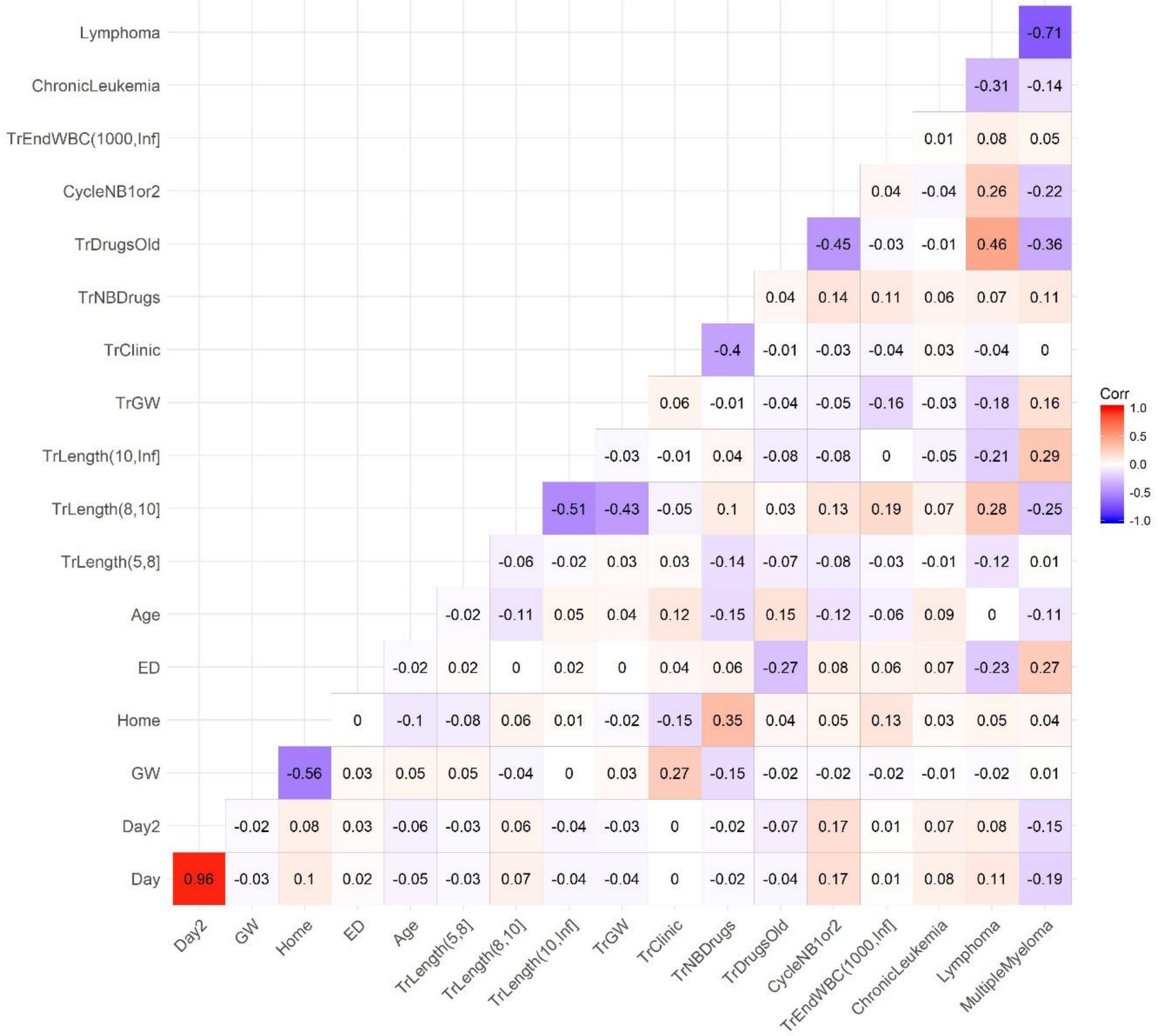


S3 Fig shows the correlation matrix for all variables in the model. As the model includes many categorical variables, generalized variance inflation factors (VIFs) have been calculated, and are displayed in S3 Table F. These generalized VIFs are the VIFs, corrected by the number of degrees of freedom (DF) of the predictor variable and may be compared to thresholds of $10^{\frac{1}{2*DF}}$ to assess collinearity (Fox & Monette, 1992). Some variables that are included as higher-order terms or in interaction terms have high VIF values, as expected. Overall, multicollinearity seems to be of little concern in the model.

**Table F. Degrees of freedom (DF) of the predictor variables for the model of infection after protocol completion analysis**

| | GVIF | Df | $10^{\frac{1}{2*DF}}$ | | GVIF | Df | $10^{\frac{1}{2*DF}}$ |
|---|---|---|---|---|---|---|---|
| DayNB | 12.487 | 1 | 3.162 | CycleNB1or2 | 1.055 | 1 | 3.162 |
| DayNB2 | 11.837 | 1 | 3.162 | TrEndWBC | 2.177 | 1 | 3.162 |
| LocStartDay | 1.202 | 2 | 1.778 | Disease | 2.692 | 3 | 1.468 |
| Age | 1.075 | 1 | 3.162 | DayNB:TrEndWBC | 13.627 | 1 | 3.162 |
| TrLength | 1.053 | 3 | 1.468 | DayNB2:TrEndWBC | 12.320 | 1 | 3.162 |
| TrLoc | 1.198 | 2 | 1.778 | DayNB:Disease | 6.404 | 3 | 1.468 |
| TrNBDrugGroups | 1.471 | 1 | 3.162 | DayNB2:Disease | 4.997 | 3 | 1.468 |
| TrDrugs | 1.122 | 1 | 3.162 | | | | |
