# Supplementary material for: The role of specialized hospital units in infection and mortality risk reduction among patients with hematological cancers
Source: PLoS One. 2019 Mar 20;14(3):e0211694. doi: 10.1371/journal.pone.0211694 (PMC6426175; doi:10.1371/journal.pone.0211694)
Supplement: S2 File — Table A. Comparison of models for mortality after infection analysis: Probit, logit, and cloglog Table B. The location as an explanatory variable in the model for mortality after infection analysis Table C. Cycle number in the model for mortality after infection analysis Table D. White blood cell counts in the model for mortality after infection analysis Table E. Type of infection in the model for mortality after infection analysis Table F. Time until antibiotic administration in the model of mortality after infection analysis Table G. Degrees of freedom (DF) of the predictor variables for the model of mortality after infection analysis Fig A. Time until antibiotic administration as a function of the location where the infection started and outcome (death) Fig B. Multicollinearity checks and variance inflation factors (VIFs). (PDF) [file pone.0211694.s004.pdf]

S4 file. Mortality after infection: model selection and sensitivity analysis

Table A. Comparison of models for mortality after infection analysis: Probit, logit, and cloglog

The final model is a probit model. The table below compares this model (on the left) with a logit or cloglog model. As the probit model has the lowest BIC value, this was chosen.

	Chosen model (probit)	Logit	Cloglog
Intercept	-3.6527 (.1517)***	-8.0763 (.4001)***	-8.0470 (.3955)***
StartHW	-.3634 (.0835)***	-.9695 (.2233)***	-.9619 (.2218)***
StartGW	.0635 (.1089)	.2109 (.2735)	.2120 (.2702)
AdmDirectHosp	-.3118 (.1243)*	-.7968 (.3298)*	-.7868 (.3271)*
AdmClinic	-.2077 (.0771)**	-.5125 (.2032)*	-.5059 (.2017)*
FirstHospGW	-.2248 (.0971)*	-.5172 (.2497)*	-.5088 (.2472)*
NoHosp	.5498 (.1660)***	1.4007 (.3953)***	1.3926 (.3854)***
GW	.1787 (.0914)	.4081 (.2322)	.3977 (.2296)
Home	-.2817 (.1306)*	-.7518 (.3525)*	-.7498 (.3495)*
ED	.2886 (.1500)	.8234 (.3684)*	.8289 (.3613)*
Day	.0583 (.0121)***	.1523 (.0309)***	.1505 (.0305)***
Day2	-.0012 (.0004)**	-.0031 (.0011)**	-.0030 (.0011)**
Age	.0124 (.0018)***	.0330 (.0047)***	.0327 (.0047)***
CycleNB	.0091 (.0035)**	.0217 (.0081)**	.0214 (.0079)**
Chemo	.2099 (.0732)**	.5424 (.1900)**	.5360 (.1879)**
InfNB	.0296 (.0082)***	.0698 (.0203)***	.0685 (.0200)***
StartWBC(2000,15000]	-.0267 (.0559)	-.0901 (.1467)	-.0903 (.1454)
StartWBC(15000,Inf]	.2610 (.0710)***	.6025 (.1776)***	.5926 (.1752)***
AIC	3040.1213	3041.9857	3042.0204
BIC	3189.7022	3191.5666	3191.6013
Log Likelihood	-1502.0606	-1502.9928	-1503.0102
Deviance	3004.1213	3005.9857	3006.0204
Num. obs.	30033	30033	30033
Coefficients marked with * indicate p<0.05; ** indicate p<0.01; *** indicate p<0.001.			

The location as an explanatory variable

The mortality model has even more information about the patient's location as explanatory variables than the previous infections model. The chosen model about where the infection started (at home, in the HW or in the GW (*StartHW* and *StartGW*), through which hospital departments the patient is admitted and hospitalized *(AdmDirectHosp, AdmClinic, FirstHospGW, NoHosp*), and where the patient resides on each day that he has an infection (In the HW, the *GW*, *home* or in the *ED*). The table below shows that a model with only location variables performs worse. A model without the location variables had a comparable BIC value and a slightly worse AIC value. We chose to keep the location variable since they add quite a lot of interesting insights.

Table B. The location as an explanatory variable in the model for mortality after infection analysis

	Chosen model	Only location	No location
Intercept	-3.6527 (.1517)***	-2.2130 (.0594)***	-3.8388 (.1448)***
StartHW	-.3634 (.0835)***	-.3484 (.0764)***	
StartGW	.0635 (.1089)	.0588 (.1098)	
AdmDirectHosp	-.3118 (.1243)*	-.2332 (.1144)*	
AdmClinic	-.2077 (.0771)**	-.1733 (.0722)*	
FirstHospGW	-.2248 (.0971)*	-.1589 (.0979)	
NoHosp	.5498 (.1660)***	.4642 (.1598)**	
GW	.1787 (.0914)	.1706 (.0922)	
Home	-.2817 (.1306)*	-.4139 (.1219)***	
ED	.2886 (.1500)	.1210 (.1388)	
Day	.0583 (.0121)***		.0479 (.0109)***
Day2	-.0012 (.0004)**		-.0010 (.0004)*
Age	.0124 (.0018)***		.0147 (.0017)***
CycleNB	.0091 (.0035)**		.0106 (.0033)**
Chemo	.2099 (.0732)**		.0920 (.0692)
InfNB	.0296 (.0082)***		.0349 (.0075)***
StartWBC(2000,15000]	-.0267 (.0559)		.0622 (.0526)
StartWBC(15000,Inf]	.2610 (.0710)***		.3490 (.0679)***
AIC	3040.1213	3207.1021	3113.5736
BIC	3189.7022	3290.2026	3188.3641
Log Likelihood	-1502.0606	-1593.5510	-1547.7868
Deviance	3004.1213	3187.1021	3095.5736
Num. obs.	30033	30033	30033
Coefficients marked with * indicate p<0.05; ** indicate p<0.01; *** indicate p<0.001.			

Table C. Cycle number in the model for mortality after infection analysis

The chosen model includes the cycle number as a continuous variable. With a BIC value of 3189.7, it outperforms the models below which include several alternative options. The first three alternative models below include a dummy variable that is 1 if it is the patient's 1st, 2nd or 1st or 2nd cycle respectively. The last model below includes CycleNB² in addition to the continuous cycle number.

	**CycleNB1**	**CycleNB2**	**1 or 2**	**Quadratic**
Intercept	-3.6702 (.1515)***	-3.6688 (.1514)***	-3.6637 (.1515)***	-3.6506 (.1518)***
StartHW	-.3520 (.0841)***	-.3777 (.0834)***	-.3582 (.0836)***	-.3600 (.0836)***
StartGW	.0690 (.1086)	.0699 (.1086)	.0655 (.1087)	.0616 (.1090)
AdmDirectHosp	-.3265 (.1242)**	-.3206 (.1240)**	-.3249 (.1243)**	-.3103 (.1244)*
AdmClinic	-.2159 (.0770)**	-.2141 (.0771)**	-.2151 (.0771)**	-.2067 (.0771)**
FirstHospGW	-.2205 (.0967)*	-.2173 (.0967)*	-.2234 (.0967)*	-.2270 (.0972)*
NoHosp	.5468 (.1656)***	.5457 (.1659)**	.5415 (.1656)**	.5451 (.1661)**
GW	.1802 (.0911)*	.1743 (.0911)	.1786 (.0912)	.1799 (.0915)*
Home	-.2729 (.1301)*	-.2796 (.1303)*	-.2756 (.1302)*	-.2789 (.1306)*
ED	.2941 (.1495)*	.2870 (.1495)	.2905 (.1496)	.2903 (.1501)
Day	.0585 (.0121)***	.0578 (.0121)***	.0583 (.0121)***	.0582 (.0121)***
Day2	-.0012 (.0004)**	-.0011 (.0004)**	-.0012 (.0004)**	-.0011 (.0004)**
Age	.0126 (.0018)***	.0127 (.0018)***	.0127 (.0018)***	.0124 (.0018)***
Chemo	.2891 (.0712)***	.2737 (.0704)***	.3057 (.0724)***	.1886 (.0769)*
InfNB	.0293 (.0083)***	.0319 (.0080)***	.0284 (.0083)***	.0285 (.0083)***
StartWBC(2000,15000]	-.0240 (.0558)	-.0254 (.0558)	-.0259 (.0558)	-.0272 (.0560)
StartWBC(15000,Inf]	.2597 (.0709)***	.2553 (.0708)***	.2570 (.0709)***	.2607 (.0710)***
1st Cycle	-.1224 (.0714)			
2nd Cycle		-.0713 (.0800)		
1st or 2nd Cycle			-.1233 (.0587)*	
continuous CycleNB				.0144 (.0067)*
Quadratic cycleNB				-.0001 (.0001)
AIC	3042.9579	3045.1884	3041.4970	3041.2557
BIC	3192.5388	3194.7693	3191.0780	3199.1467
Log Likelihood	-1503.4789	-1504.5942	-1502.7485	-1501.6279
Deviance	3006.9579	3009.1884	3005.4970	3003.2557
Num. obs.	30033	30033	30033	30033
Coefficients marked with * indicate p<0.05; ** indicate p<0.01; *** indicate p<0.001.				

The choice for how the WBC counts were split up into different categories was inspired by physicians' experience but several other models with other choices for these categories were tested but rejected because they had a higher BIC value. The table below shows one alternative choice for the WBC as a categorical variable. The third and fourth models in the table add the WBC (and WBC^2) as a continuous variable; both have higher BIC values.

Table D. White blood cell counts in the model for mortality after infection analysis

	**Chosen model**	**Categorical WBC**	**Continuous WBC**	**Continuous and quadratic WBC**
Intercept	-3.6527 (.1517)***	-3.6036 (.1549)***	-3.7094 (.1514)***	-3.7308 (.1521)***
StartHW	-.3634 (.0835)***	-.3747 (.0839)***	-.3562 (.0825)***	-.3441 (.0834)***
StartGW	.0635 (.1089)	.0628 (.1087)	.0666 (.1090)	.0732 (.1094)
AdmDirectHosp	-.3118 (.1243)*	-.3000 (.1245)*	-.3034 (.1235)*	-.3002 (.1237)*
AdmClinic	-.2077 (.0771)**	-.2007 (.0771)**	-.2159 (.0767)**	-.2072 (.0771)**
FirstHospGW	-.2248 (.0971)*	-.2254 (.0970)*	-.2241 (.0977)*	-.2177 (.0980)*
NoHosp	.5498 (.1660)***	.5567 (.1662)***	.5312 (.1655)**	.5362 (.1657)**
GW	.1787 (.0914)	.1832 (.0912)*	.1912 (.0916)*	.1865 (.0919)*
Home	-.2817 (.1306)*	-.2793 (.1305)*	-.2840 (.1313)*	-.2818 (.1312)*
ED	.2886 (.1500)	.2940 (.1502)	.3077 (.1497)*	.3020 (.1501)*
Day	.0583 (.0121)***	.0586 (.0121)***	.0588 (.0121)***	.0588 (.0121)***
Day2	-.0012 (.0004)**	-.0011 (.0004)**	-.0012 (.0004)**	-.0012 (.0004)**
Age	.0124 (.0018)***	.0126 (.0018)***	.0129 (.0018)***	.0128 (.0018)***
CycleNB	.0091 (.0035)**	.0089 (.0035)*	.0085 (.0035)*	.0085 (.0035)*
Chemo	.2099 (.0732)**	.2061 (.0736)**	.2274 (.0739)**	.2354 (.0743)**
InfNB	.0296 (.0082)***	.0298 (.0082)***	.0302 (.0082)***	.0300 (.0082)***
StartWBC(2000,15000]	-.0267 (.0559)			
StartWBC(15000,Inf]	.2610 (.0710)***			
StartWBC(500,1000]		-.1392 (.1029)		
StartWBC(1000,15000]		-.0903 (.0693)		
StartWBC(15000,Inf[		.1969 (.0842)*		
WBC			.0000 (.0000)***	.0000 (.0000)**
Quadratic WBC				-.0000 (.0000)
AIC	3040.1213	3039.9647	3042.0284	3042.2914
BIC	3189.7022	3197.8557	3183.2993	3191.8723
Log Likelihood	-1502.0606	-1500.9823	-1504.0142	-1503.1457
Deviance	3004.1213	3001.9647	3008.0284	3006.2914
Num. obs.	30033	30033	30033	30033
Coefficients marked with * indicate p<0.05; ** indicate p<0.01; *** indicate p<0.001.				

Type of infection

Since we had information on the type of drugs that were administered and the ICD9 codes for many infections, we could distinguish between viral, bacterial and fungal infections. There wasn't one particular type of infection, though, that had significantly higher or lower infection probabilities. They were left out in the chosen model.

Table E. Type of infection in the model for mortality after infection analysis

	Chosen model	Bacterial	Fungal	Viral	All
Intercept	-3.6527 (.1517)***	-3.7143 (.1659)***	-3.6552 (.1519)***	-3.6476 (.1516)***	-3.7707 (.1852)***
StartHW	-.3634 (.0835)***	-.3658 (.0834)***	-.3569 (.0840)***	-.3634 (.0834)***	-.3554 (.0838)***
StartGW	.0635 (.1089)	.0590 (.1089)	.0781 (.1105)	.0661 (.1089)	.0860 (.1101)
AdmDirectHosp	-.3118 (.1243)*	-.2818 (.1283)*	-.3480 (.1322)**	-.3082 (.1244)*	-.3304 (.1324)*
AdmClinic	-.2077 (.0771)**	-.1962 (.0778)*	-.2181 (.0782)**	-.2006 (.0771)**	-.2051 (.0783)**
FirstHospGW	-.2248 (.0971)*	-.2382 (.0980)*	-.2076 (.0992)*	-.2216 (.0972)*	-.2131 (.0990)*
NoHosp	.5498 (.1660)***	.5502 (.1658)***	.5649 (.1673)***	.5545 (.1658)***	.5831 (.1679)***
GW	.1787 (.0914)	.1813 (.0915)*	.1718 (.0916)	.1707 (.0916)	.1661 (.0913)
Home	-.2817 (.1306)*	-.2749 (.1301)*	-.2841 (.1308)*	-.2873 (.1308)*	-.2763 (.1300)*
ED	.2886 (.1500)	.2875 (.1501)	.2845 (.1501)	.2829 (.1502)	.2763 (.1502)
Day	.0583 (.0121)***	.0576 (.0121)***	.0582 (.0121)***	.0584 (.0121)***	.0570 (.0121)***
Day2	-.0012 (.0004)**	-.0011 (.0004)**	-.0012 (.0004)**	-.0012 (.0004)**	-.0011 (.0004)**
Age	.0124 (.0018)***	.0124 (.0018)***	.0124 (.0018)***	.0123 (.0018)***	.0123 (.0018)***
CycleNB	.0091 (.0035)**	.0092 (.0035)**	.0093 (.0035)**	.0095 (.0036)**	.0098 (.0035)**
Chemo	.2099 (.0732)**	.2137 (.0734)**	.2050 (.0734)**	.2121 (.0733)**	.2075 (.0735)**
InfNB	.0296 (.0082)***	.0297 (.0081)***	.0294 (.0082)***	.0298 (.0081)***	.0294 (.0082)***
StartWBC (2000,15000]	-.0267 (.0559)	-.0165 (.0572)	-.0342 (.0567)	-.0192 (.0563)	-.0214 (.0574)
StartWBC (15000,Inf]	.2610 (.0710)***	.2672 (.0715)***	.2575 (.0712)***	.2674 (.0713)***	.2672 (.0716)***
Bacterial		.0691 (.0738)			.1293 (.1130)
Fungal			.0835 (.0963)		.1780 (.1351)
Viral				-.1648 (.1130)	-.0700 (.1366)
AIC	3040.1213	3041.2116	3041.3662	3039.7994	3041.9257
BIC	3189.7022	3199.1026	3199.2572	3197.6903	3216.4368
Log Likelihood	-1502.0606	-1501.6058	-1501.6831	-1500.8997	-1499.9629
Deviance	3004.1213	3003.2116	3003.3662	3001.7994	2999.9257
Num. obs.	30033	30033	30033	30033	30033

Coefficients marked with * indicate p<0.05; ** indicate p<0.01; *** indicate p<0.001.

Time until antibiotic administration

Putt & Jones (2014) established a target of one hour door to needle for emergency oncology patients in the UK. Therefore, we wanted to check the time until the start of antibiotics treatment (*AntibTime*). For infections that start at home, the time until the start of antibiotics treatment (*AntibTime*) is estimated to be the time between the arrival of the patient to the hospital and the first antibiotics administration. For infections that start while the patient is hospitalized, estimating the time until the start of treatment is more difficult since infections are often detected in the data because of the start of antibiotics treatment (see Methodology section and S2 file) which means the start of the infection and the start of treatment coincide for these infections. Therefore, the time between the last temperature measurement that did not indicate fever and the start of the infection is used as an upper bound for *AntibTime*. The reasoning is that the nurse or physician checked the patient during that temperature check and concluded that the patient's condition was under control. S4 Fig A demonstrates the estimated time until the start of antibiotics (AntibTime) as a function of the location where the infection started and the patient outcome (death). It shows that *AntibTime* is the lowest in the HW. Adding *AntibTime* to the basic model does not significantly improve the predictive power of the model (see S4 Table F); these effects are possibly already captured by the StartHW and StartGW dummies.

Fig A. Time until antibiotic administration as a function of the location where the infection started and outcome (death)

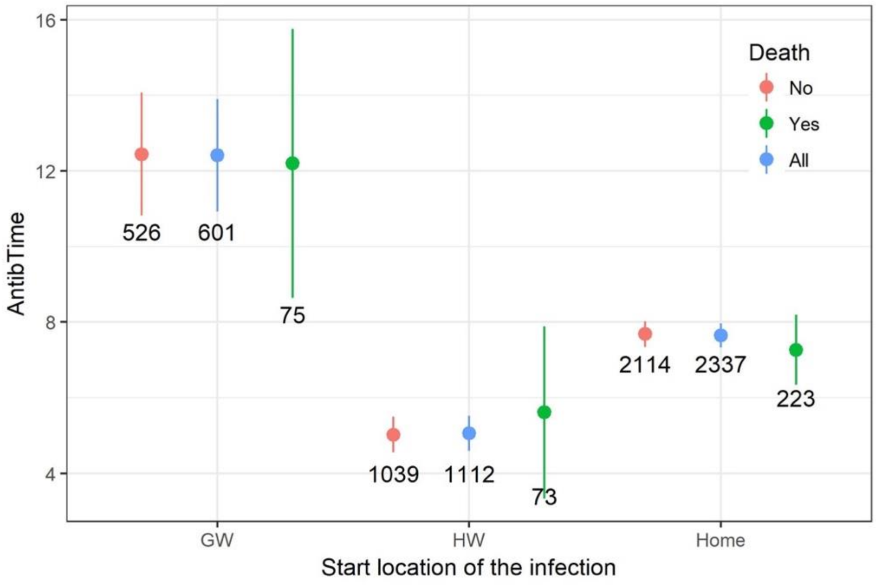

Table F. Time until antibiotic administration in the model of mortality after infection analysis

	**Chosen model**	**With time until antibiotics administration**
Intercept	-3.6527 (.1517)***	-3.7069 (.1577)***
StartHW	-.3634 (.0835)***	-.3478 (.0838)***
StartGW	.0635 (.1089)	.0834 (.1140)
AdmDirectHosp	-.3118 (.1243)*	-.3156 (.1246)*
AdmClinic	-.2077 (.0771)**	-.2047 (.0783)**
FirstHospGW	-.2248 (.0971)*	-.2213 (.0982)*
NoHosp	.5498 (.1660)***	.6914 (.1697)***
GW	.1787 (.0914)	.1726 (.0939)
Home	-.2817 (.1306)*	-.2463 (.1295)
ED	.2886 (.1500)	.2869 (.1526)
Day	.0583 (.0121)***	.0593 (.0124)***
Day2	-.0012 (.0004)**	-.0012 (.0004)**
Age	.0124 (.0018)***	.0130 (.0018)***
CycleNB	.0091 (.0035)**	.0080 (.0036)*
Chemo	.2099 (.0732)**	.2139 (.0751)**
InfNB	.0296 (.0082)***	.0307 (.0083)***
StartWBC(2000,15000]	-.0267 (.0559)	.0077 (.0568)
StartWBC(15000,Inf]	.2610 (.0710)***	.2803 (.0725)***
AntibTime		-.0417 (.0613)
AIC	3040.1213	2926.2463
BIC	3189.7022	3083.5298
Log Likelihood	-1502.0606	-1444.1231
Deviance	3004.1213	2888.2463
Num. obs.	30033	29088
Coefficients marked with * indicate p<0.05; ** indicate p<0.01; *** indicate p<0.001.		

Multicollinearity checks for the mortality after infection model

S4 Fig B shows the correlation matrix for all variables in the model. We preformed the same tests as we did for the infection model.

Fig B. Multicollinearity checks and variance inflation factors (VIFs)

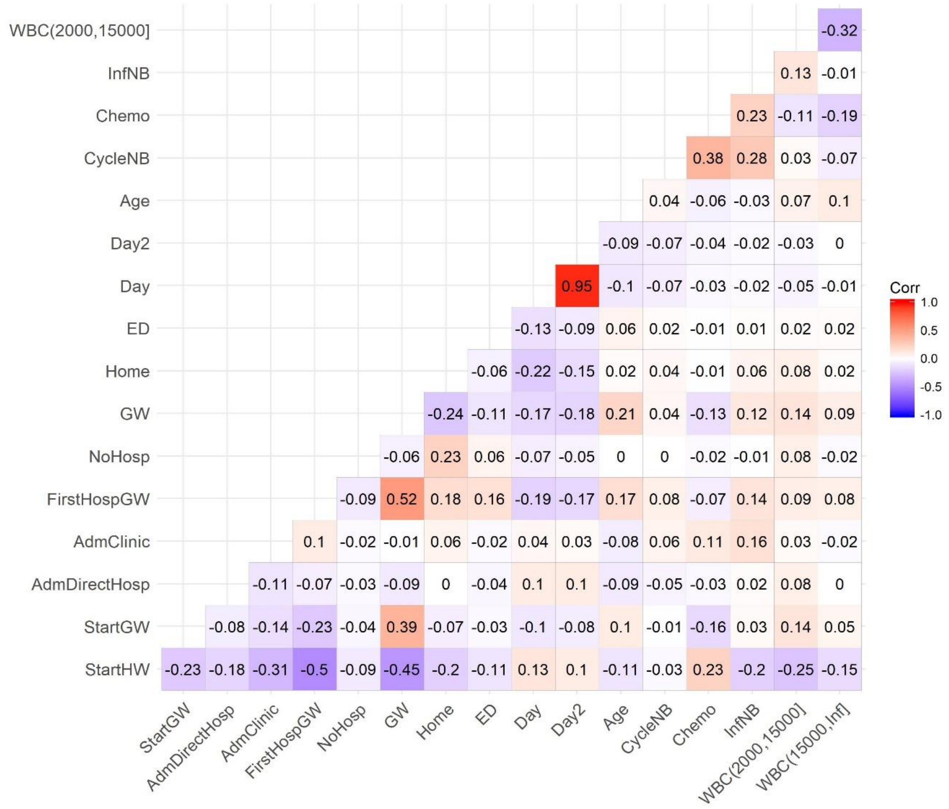

Table G. Degrees of freedom (DF) of the predictor variables for the model of mortality after infection analysis

	**GVIF**	**Df**	$10^{\frac{1}{2*DF}}$
Start location & admission (*HW, GW, Home+ED, Home+Clinic, Home+DirectHosp*)	1.534	4	1.334
First hospitalization location (*HW,GW,NoHosp*)	2.248	2	1.778
Location at the start of the day	1.300	3	1.468
DayNB	3.920	1	3.162
DayNB2	3.791	1	3.162
Age	1.096	1	3.162
CycleNB	1.090	1	3.162
Chemo	1.147	1	3.162
InfNB	1.097	1	3.162
StartWBC	1.056	2	1.778

Fox J, Monette G. (1992). Generalized collinearity diagnostics. *Journal of the American Statistical Association*, 87(417), 178–183.

Putt L, Jones P. (2014). The role of the specialist acute oncology nurse in the new acute oncology services. *Clinical Oncology*, 26(3), 125 – 127.
