# Supplementary material for: The role of specialized hospital units in infection and mortality risk reduction among patients with hematological cancers
Source: PLoS One. 2019 Mar 20;14(3):e0211694. doi: 10.1371/journal.pone.0211694 (PMC6426175; doi:10.1371/journal.pone.0211694)

S5 file. Distribution of the WBC counts among the evaluated patients

WBC counts turned out to be strong predictors for infection susceptibility in our patient cohort, as expected by the physicians. S5 Fig shows density curves for the WBC counts that are observed at the start and end of the protocol for different types of protocols and patients. These curves are based on all protocols in the dataset for which a WBC count is available, both at the beginning and the end of the protocol. The difference between the WBC count at the beginning and the end of the protocol is particularly large for AL patients, longer protocols, or protocols for which the patient was hospitalized. Patients that eventually got an infection within 30 days after the end of the protocol seem to end their protocol with lower WBC counts on average.

S5 Fig. Distribution of the WBC counts among the evaluated patients based on different factors (disease, protocol length, number of treatment cycles, hospitalization, infection occurrence, and patient age)

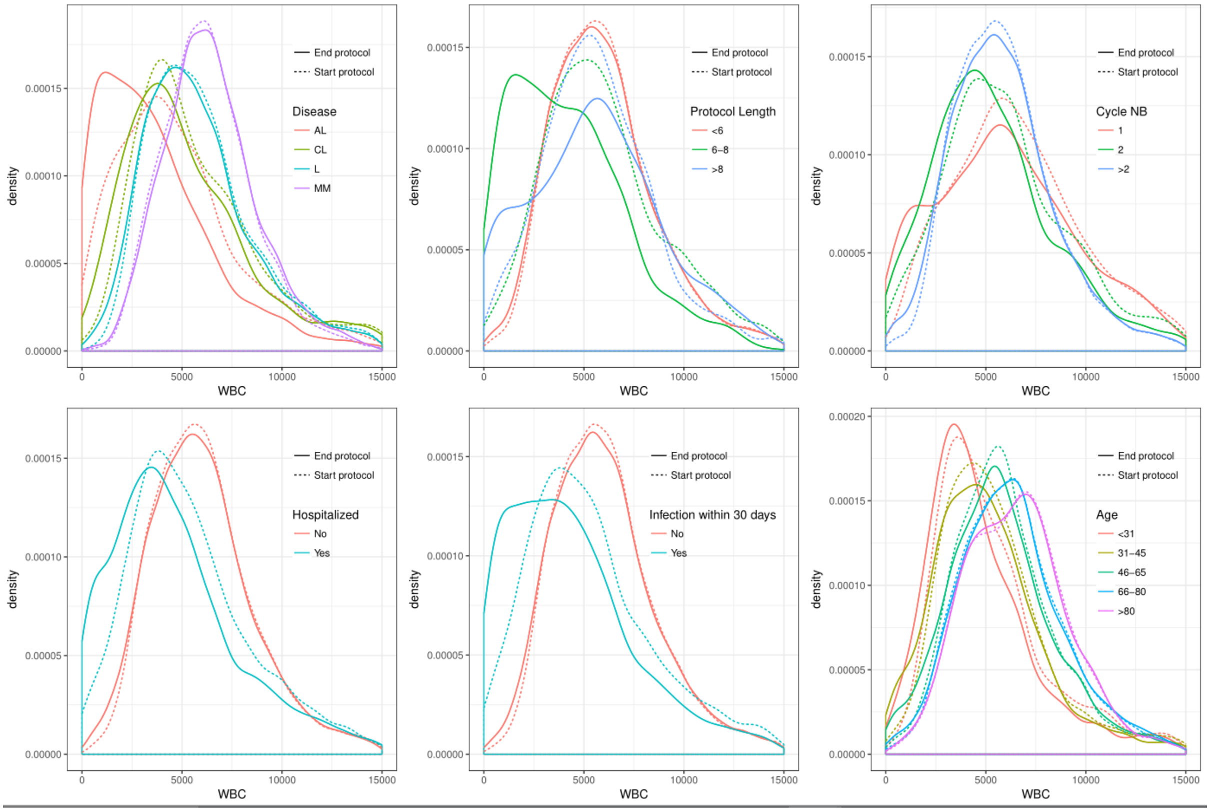

Supplement: S1 Fig — (PDF) [file pone.0211694.s005.pdf]
