# Supplementary material for: The role of specialized hospital units in infection and mortality risk reduction among patients with hematological cancers
Source: PLoS One. 2019 Mar 20;14(3):e0211694. doi: 10.1371/journal.pone.0211694 (PMC6426175; doi:10.1371/journal.pone.0211694)

<u>S6 file</u>. Per time-of-the-day analysis of the time lag between patient's emergency presentation and hospitalization

The time lag between emergency presentation and hospitalization was compared for patients treated at the ED and HOutC during the morning hours when both options were available. The time lags changed according to the time of the day.

S6 Fig shows the average and 95% confidence intervals for the hospitalization time lag for patients presenting to the hospital with an infection. Weekend arrivals are excluded.

S6 Fig. Hospitalization delay by entry gate to the hospital (ED vs. HOutC) and the hour of admission

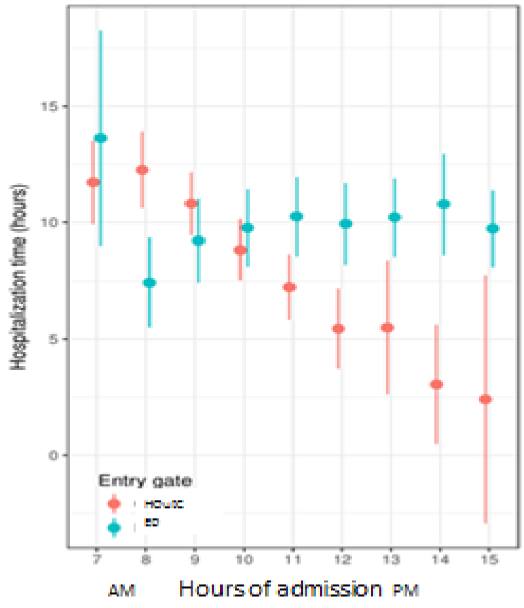

Supplement: S2 Fig — (PDF) [file pone.0211694.s006.pdf]
